# Role of Monovalent Ions in the NKCC1 Inhibition Mechanism Revealed through Molecular Simulations

**DOI:** 10.3390/ijms232315439

**Published:** 2022-12-06

**Authors:** Pavel Janoš, Alessandra Magistrato

**Affiliations:** CNR-IOM c/o International School for Advanced Studies (SISSA/ISAS), Via Bonomea 265, 34136 Trieste, Italy

**Keywords:** ion transporters, monovalent ions, molecular dynamics, drug design, enhanced sampling, membrane transporters, dynamic docking

## Abstract

The secondary active Na-K-Cl cotransporter 1 (NKCC1) promotes electroneutral uptake of two chloride ions, one sodium ion and one potassium ion. NKCC1 regulates Cl^−^ homeostasis, thus being implicated in transepithelial water transport and in neuronal excitability. Aberrant NKCC1 transport is linked to a variety of human diseases. The loop diuretic drugs bumetanide, furosemide, azosemide and ethacrynic acid target NKCC1, but are characterized by poor selectivity leading to severe side effects. Despite its therapeutic importance, the molecular details of the NKCC1 inhibition mechanism remain unclear. Using all-atom simulations, we predict a putative binding mode of these drugs to the zebrafish (z) and human (h) NKCC1 orthologs. Although differing in their specific interactions with NKCC1 and/or monovalent ions, all drugs can fit within the same cavity and engage in hydrophobic interactions with M304/M382 in z/hNKCC1, a proposed ion gating residue demonstrated to be key for bumetanide binding. Consistent with experimental evidence, all drugs take advantage of the K^+^/Na^+^ ions, which plastically respond to their binding. This study not only provides atomic-level insights useful for drug discovery campaigns of more selective/potent NKCC1 inhibitors aimed to tackle diseases related to deregulated Cl^−^ homeostasis, but it also supplies a paradigmatic example of the key importance of dynamical effects when drug binding is mediated by monovalent ions.

## 1. Introduction

The cation-chloride cotransporter (CCC) protein family is composed of two sodium–potassium–chloride cotransporters (NKCC1-2) and four Na^+^-independent cotransporters (KCC1-4). The NKCC transporters translocate Cl^−^ ions in/out of the plasma membrane of distinct cell types [1,2]. By exploiting the K^+^ and/or Na^+^ concentration gradient, generated by the Na^+^–K^+^–ATPase [1,3,4], NKCC transport the Na^+^, K^+^, and Cl^−^ ions in a 1:1:2 stoichiometry [5] across cellular membranes, thus maintaining intracellular Cl^−^ concentration beyond its electrochemical equilibrium. Conversely, the KCCs extrude Cl^−^ ions from the cell following an outwards K^+^ gradient. The tight and reciprocal regulation of NKCC(s) and KCC(s) by kinases and phosphatases is critical in maintaining intracellular Cl^−^ homeostasis. Among the two NKCC isoforms, the NKCC1 is expressed in all tissues, while NKCC2 is found exclusively in kidneys. Owing to its ubiquitous distribution across the human body, NKCC1 plays key roles in a multitude of biological processes such as: (i) salt reabsorption in the basolateral membrane of exocrine glands; (ii) maintenance of the endolymph fluid present in the inner part of the ear required for hearing; and (iii) regulation of neuronal excitability and γ-aminobutyric acid-mediated signaling [6].

**Figure 1:**
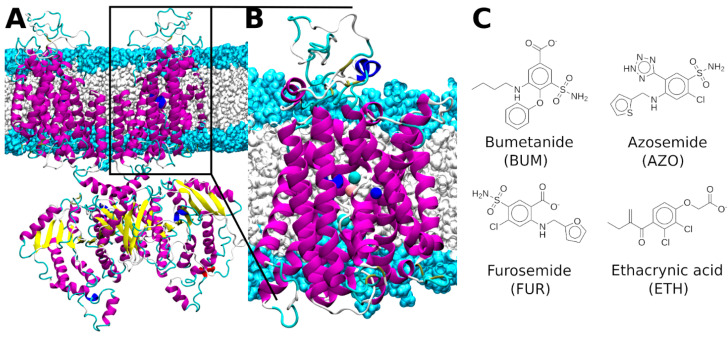
(**A**) Full length dimeric structure of zebrafish (z)NKCC1. (**B**) The monomeric transmembrane model of zNKCC1. NKCC1 is depicted as magenta, yellow, blue, cyan, and white new cartoons for α-helix, β-sheet, 3_10_-helix, turn and coil elements, respectively. The Cl^−^, Na^+^, and K^+^ ions are shown as cyan, blue, and orange van der Waals spheres, respectively. The hydrophilic head groups and hydrophobic tails of the phosphatidylcholine lipids are depicted in light blue and white surfaces, respectively. (**C**) Sketches of the four investigated NKCC1 inhibitors.

Unsurprisingly, an altered NKCC1 transport is implicated in several diseases: neonatal seizures, neuropathic pain, and neurological disorders (including Down syndrome and autism spectrum disorders) [7,8,9], hypertension [10] and renal disturbances (Gitelman’s, Bartter’s, and Andermann’s syndromes) [11]. Moreover, NKCC1 expression is also linked to tumor infiltration and progression in multiple cancer types (lung adenocarcinoma, prostate cancer, esophageal squamous carcinoma, and brain tumors) [12]. As such, NKCC1 is an emerging pharmacological target against Cl^−^ dishomeostasis-related diseases. The so-called loop diuretic drugs, furosemide (FUR) and bumetanide (BUM), inhibit NKCC transporters. These are commonly prescribed for pulmonary edema [13], but they also exhibit antiseizure [14], antiepileptic [15], antiparkinsonian [16], and antitumor activity [12]. Bumetanide (BUM) is the most studied NKCC inhibitor (Figure 1C), with a plethora of experimental data documenting its inhibitory effect [17,18,19,20], and the only loop diuretic to show some level of selectivity for NKCC1 [21,22]. BUM reaches an IC_50_ of ~1.0 μM [23] and therapeutic potency in the ~2–10 μM range [24]. It has also been shown that BUM (and other loop diuretics) competes with the binding of one of the co-transported Cl^−^ ions [3,18], while it requires the presence of the other monovalent ions for binding and inhibition of the ion transport [3,17]. Mutagenesis experiments identified a series of NKCC1 residues potentially involved in BUM binding. These are located along the Transmembrane helix 3 (TM3) and include the M304/M382 residue, which was proposed to serve as an ion-gating amino acid [25]. The intracellular import of BUM is facilitated by Monocarboxylate Transporter 6 (MCT6) [26,27] and its export from cells is mediated by three transporters: Multidrug Resistance Protein 4 (MRP4), Organic Anion Transporter 3 (OAT3) and Organic Anion Transporting Polypeptide 2 (OATP2) [28]. Indeed, the inhibition of their export has been proposed as a therapeutic strategy to increase the amount of available intracellular BUM [29]. Despite the promising results in using BUM for treating epilepsy and other brain disorders [30], two issues preclude its effectiveness: (i) poor brain penetration due to its negative charges at physiological pH and its high plasma protein binding [31], and (ii) its still poor selectivity between NKCC1 and NKCC2 isoforms, which leads to a number of adverse side effects such as diuresis, hypokalemic alkalosis, and hearing loss [32]. Furosemide (FUR) (Figure 1C), a sulfamoyl benzoic acid derivative of BUM, is the second clinically-prescribed NKCC inhibitor. FUR, however, exhibits ~40-fold lower therapeutic potency than BUM [19,20] with a IC_50_ of ~5–6 μM [23]. Azosemide (AZO) (Figure 1C), a non-acidic sulfamoyl derivative of BUM, is more potent than BUM (IC_50_ ~0.2 µM) and has better tissue distribution and transport properties [23]. Conversely, ethacrynic acid (ETH) (Figure 1C) is the least potent among the available NKCC inhibitors (IC_50_ ~1.6–3.0 mM) [23]. Nevertheless, lacking the sulfamoyl moiety, it can be prescribed to patients with sulfa allergies [33]. Similar to other loop diuretics, it still exhibits ototoxic side effects [34].

The NKCC1 structure is composed of two inverted repeats of five transmembrane helices arranged in a pseudosymmetric topology forming a central binding cavity. NKCC1 is a homodimeric protein. Each monomer contains a transmembrane domain, responsible for the ion transport, and N-terminal and C-terminal domains, which, beside contributing to dimer assembly, possibly modulate ion trafficking (Figure 1A) [35,36]. NKCC1 ion transport proceeds via a so-called alternating-access mechanism, whereby the transporter visits an outward-facing, occluded, and inward-facing conformation [37,38,38,39].

The first cryo-EM structure of dimeric zebrafish NKCC1 (zNKCC1) [35] trapped the transporter in its inward-facing state (Figure 1) and also traced two Cl^−^ and one K^+^ ions in their binding sites. The position of the Na^+^ binding site was, instead, inferred based on the structural homology with other transporters. Previously, we used this zNKCC1 inward-facing structure to establish the ion transport mechanism of zNKCC1. Taking advantage of this zNKCC1 structure, and of a more recent human (h)NKCC1 structure [36], here we characterized the potential binding modes of the loop diuretic inhibitors. Combined static and dynamic (metadynamics-based) docking simulations enabled us not only to assess a putative loop-diuretics binding mode to the z/hNKCC1, but also to uncover the key role of monovalent metal ions plasticity and adaptability in mediating and stabilizing their binding.

## 2. Results

### 2.1. Bumetanide Binding Pose Refinement

**Figure 2:**
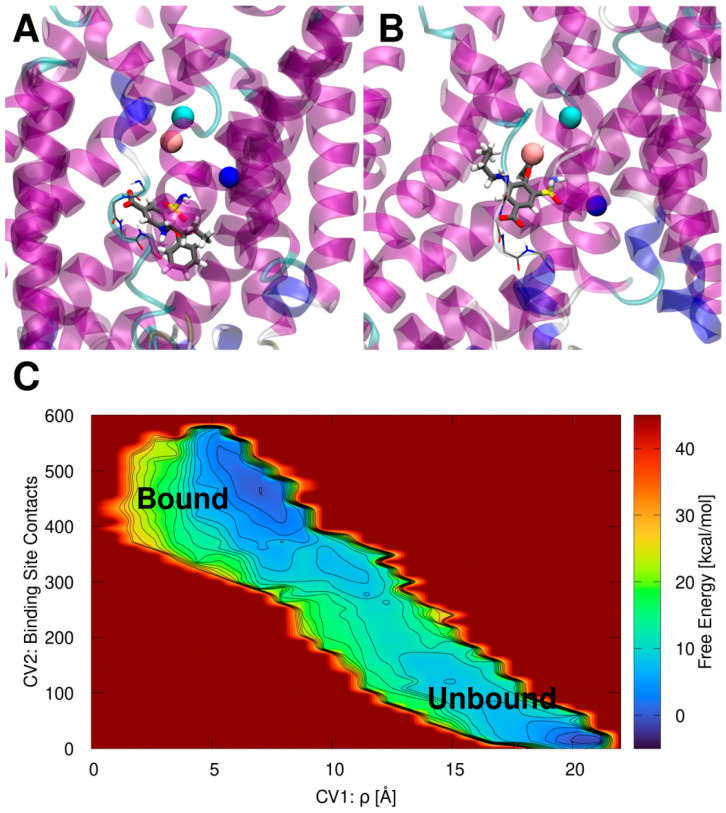
(**A**) Bumetanide (BUM) binding pose inside the zebrafish (z)NKCC1 structure as obtained from docking simulation. (**B**) BUM binding pose inside zNKCC1 as obtained from metadynamics simulation. NKCC1 is depicted as as magenta, blue, cyan, and white new cartoons for α-helix, 3_10_-helix, turn and coil elements, respectively. The Cl^−^, Na^+^, and K^+^ ions are shown as cyan, blue, and orange van der Waals spheres, respectively. The BUM is shown in licorice and colored by atom name. The (empty) bCl^−^ binding site is shown as thin licorice for visual reference. (**C**) Free energy surface (kcal/mol) of the BUM binding/dissociation extracted from the volume-based metadynamics simulation of the ion-bound (holo) zNKCC1. The free energy is plotted as a function of the ligand center of mass with respect to the center of mass of the protein CV1 ρ (Å) and the number of contacts of BUM with its binding site (CV2). Free energy is reported in color ranging from dark blue to dark red. Contour lines are reported every 2.0 kcal/mol.

First, we simulated the binding of BUM to zNKCC1 since this is the most studied NKCC1 inhibitor. The initial BUM binding pose obtained from docking simulations (Figure 2A) was refined using volume-based metadynamics (vMTD) simulations to enhance the dissociation and binding processes of the drug. This allowed us to explore and rank at the same time several possible BUM binding modes within NKCC1. The vMTD simulations were performed both in the presence and in the absence of the K^+^ and Na^+^ ions, and the more inner buried Cl^−^, hereafter named top (t)Cl^−^ ion (holo and apo NKCCK1, respectively). The other more cytosol exposed Cl^−^ ion, originally trapped in the cryoEM structure (named as “bottom” (b)Cl^−^), was removed. Indeed, the experimental evidence indicates that BUM binding competes with Cl^−^ binding [3,18]. These experimental data are in line with our previous study on the zNKCC1 ion transport mechanism, where we showed that bCl^−^ is the most weakly bound ion and therefore it is the most likely to leave NKCC1 first [40]. Moreover, when docking BUM, its carboxylic group was placed inside the binding site of bCl^−^. Therefore, it is reasonable to assume that BUM “out-competes” the bCl^−^ ion specifically. A comparison of the vMTD simulations assessing the binding modes of BUM to the apo (Figure S1; Table S1) and to the ion-occupied (holo) (Figure S2; Table S2) NKCC1 forms reveals that in the apo model the (un)binding free energy surface (FES) is very shallow and presents several metastable minima. Here the deepest minimum (the most stable BUM binding pose) corresponds to the dissociated state of BUM. Conversely, the state corresponding to the drug bound inside the zNKCC1 cavity is only the third most stable minimum (Figure S1; Table S1). As such, the apo MTD simulation results in an unfavorable (positive) binding free energy (ΔG_b_) of 13.6 kcal/mol of BUM, while its dissociation free energy barrier (ΔG_d_^‡^) is of 10 kcal/mol. This suggests that the binding of BUM to the apo zNKCC1 is disfavored. Conversely, the FES resulting from the vMTD simulation for the BUM binding to holo zNKCC1 (Figure S2; Table S2) exhibits a well-defined minimum corresponding to the BUM bound state. This is characterized by ΔG_d_^‡^ of 10 kcal/mol and a ΔG_b_ of −3.5 kcal/mol. Taken together these results clearly indicate that BUM binds to zNKCC1 only in the presence of K^+^, Na^+^ and tCl^−^ ions, in agreement with experimental data [3,17].

Notably, in the initial docking pose (Figure 2A) the carboxyl group of BUM occupies the bCl^−^ site and its hydrophobic parts are oriented towards the zNKCC1 exit channel. However, after refinement by the vMTD simulation (Figure 2B), BUM sits deeper in the zNKCC1 with its aliphatic chain and phenyl ring oriented towards the zNKCC1 interior. Even in this pose the carboxyl group occupies the bCl^−^ binding site, engaging in H-bond interactions with the backbone NH atoms of I422 and L423, which, when present, coordinate the bCl^−^ ion [40]. Conversely, the BUM sulfamoyl moiety interacts with the Na^+^ and K^+^ ions (Figure 2B).

**Figure 3:**
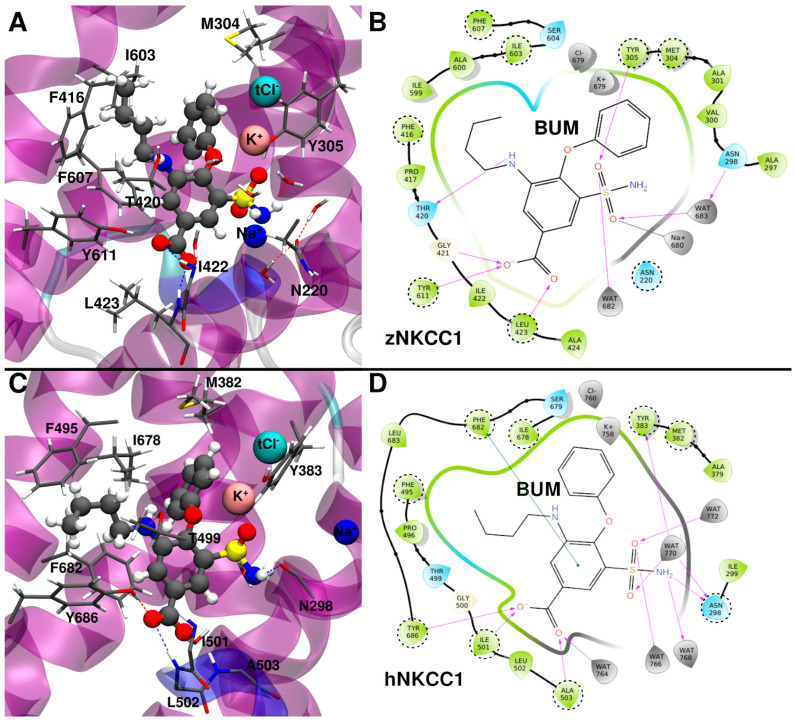
(**A**) Binding mode of bumetanide (BUM) to zebrafish (z)NKCC1 taken from a representative structure of the most populated cluster extracted from the molecular dynamics (MD) trajectory. (**B**) 2D schematic representation of the BUM/zNKCC1 interactions. (**C**) Binding mode of BUM to human (h)NKCC1 taken from a representative structure of the most populated cluster extracted from the MD trajectory. (**D**) 2D schematic representation of the BUM/hNKCC1 interactions. Polar/charged protein residues are colored blue, while the others are colored green. Waters and ions are colored gray. The most important protein residues, identified by alanine scanning analysis, are highlighted by dashed circles.

To further assess the reliability and the stability of the vMTD-refined binding pose of BUM, we performed a 500 ns-long MD simulation of the BUM/zNKCC1 complex. This allowed us to gain enough statistics and to better annotate the interactions that BUM establishes with the cytosolic cavity of the transporter (Figure 3A,B). Even after this long and unbiased MD simulation BUM stably (Figure S3) binds within zNKCC1, retaining the full set of the initial interactions (Figure 3A,B). Yet, the ions rearrange slightly during the MD simulation. Briefly, (i) the K^+^ ion undergoes small adjustment, but remains stably bound in its binding site, (ii) the tCl^−^ ion slightly moves, but it remains held by the K^+^ ion; (iii) the Na^+^ ion departs from its binding site and becomes partially coordinated by the BUM sulfamoyl group.

Next, by performing in silico alanine scanning analysis, we pinpointed the residues that more strongly contribute to BUM binding (Table S3). As a result, we observed that F607, I603 and F416 engage in hydrophobic interactions with the BUM aliphatic chains, while M304 and Y305 establish hydrophobic interactions with its phenyl ring. Conversely, the BUM sulfamoyl group forms direct and water-mediated H-bonds with Y305 and N220, respectively. A visual inspection also reveals that the key stabilizing interactions are the H-bonds between the BUM carboxylic group and the backbone NH of I422 and L423, that form the bCl^−^ binding site. We ultimately calculated the ΔG_b_ without considering each ion in order to assess their individual impact on the BUM binding (Table S3). As a result, we observed that the K^+^ and Na^+^ ions provide a stabilizing effect, though K^+^ to a larger degree, while Cl^−^ slightly destabilizes BUM due to the electrostatic repulsion. Notably, the importance of M304 residue, observed in our simulation, is fully consistent with mutagenesis studies. This supports the reliability of our predicted binding pose [25].

### 2.2. Bumetanide Binding to Human NKCC1

Before investigating the binding of BUM to hNKCC1, we inspected the ions stability and their dynamics in the model of the human structure. To this end we performed 500 ns-long MD simulation of the fully ion-loaded (Na^+^, K^+^, two Cl^−^) hNKCC1 model and compared the ions’ behaviors with those observed in the MD simulation of zNKCC1 (Figures S4 and S5) [40]. As a result, the K^+^ and tCl^−^ remained stably bound within their binding sites, similar to what we observed for zNKCC1. The bCl^−^ was the most mobile of all ions in both the z and hNKCC1 simulations, but its mobility was larger in hNKCC1. This ion partially dissociated from its binding site but came back multiple times, and finally completely dissociated from hNKCC1. This behavior is consistent with our previous results showing that bCl^−^ is the first ion to leave zNKCC1 [40]. Interestingly, even Na^+^ is more mobile in hNKCC1 than in zNKCC1, undergoing substantial remodeling without dissociating completely. The increased mobility of both bCl^−^ and Na^+^ in hNKCC1 as compared to zNKCC1 is noteworthy and consistent with a recent ion transport study of hNKCC1 [41].

Next, to verify the relevance of the BUM binding pose, identified using the zNKCC1 model, even for the human ortholog, we used the holo hNKCC1 structure previously equilibrated in MD simulation and we docked the drug into the same binding pose as in the zNCC1 model. The resulting BUM/hNKCC1 complex was relaxed by performing 500 ns-long MD simulations (Figure 3C,D). Interestingly, BUM remained stably bound in the same binding pose found in zNKCC1 (Figure S6). Its carboxylic group stayed in the bCl^−^ binding site, the aliphatic chain and the phenyl ring fitted inside an NKCC1 hydrophobic cavity, while the sulfamoyl group was partially coordinated by the K^+^ ion. The K^+^, Na^+^ tCl^−^ ions stayed in their binding cavities. Conversely, tCl^−^ rearranged, but remained bound to the hNKCC1 thanks to the electrostatic stabilization/structural trapping exerted by the K^+^ ion. At variance to what we observed in the zNKCC1/BUM complex, the Na^+^ ion remained within its binding site.

Interestingly, BUM exhibits a lower ΔG_b_ towards hNKCC1 as compared to zNKCC1 (−26.4 ± 4.7 kcal/mol vs. −21.7 ± 3.9 kcal/mol, Table 1). Similar to zNKCC1, the alanine scanning analysis confirms that F682, F495 and I678 strongly contribute to BUM binding by forming a hydrophobic pocket where the BUM aliphatic chain fits; in addition to M382 and Y383, which establish hydrophobic and π-stacking interactions with the inhibitor phenyl ring. Moreover, Y686 and the backbone NH atoms of the I501 and L502 H-bond with the BUM carboxyl group. The larger number of interactions that BUM establishes in the human as compared to the zebrafish ortholog explain the difference in ΔG_b_. The main difference stems from the fact that in hNKCC1 BUM H-bonds to Y686 (Y611 in zNKCC1) side chain, while this interaction is absent in the BUM/zNKCC1 complex. The contribution of K^+^ and tCl^−^ to BUM ΔG_b_ (Table S4) is similar in the two orthologs. While that of Na^+^ was smaller in hNKCC1 since here BUM does not coordinate this ion as in zNKCC1. Consistently with previous observations, the M382 (M304 in zNKCC1) strongly stabilizes BUM binding to hNKCC1.

In order to chase-down the source of the differences between zNKCC1 and hNKCC1 in the ions and drug binding mode we performed sequence (Figure S7) and structural alignment of the two orthologs (Figure S8). Notably, the transmembrane helices (TM1-TM12) show a remarkable level of sequence conservation (Figure S7), while the loops connecting the TMs exhibit some sequence differences. By inspecting their structural alignment (Figure S8A), it clearly emerges that the largest differences, beyond the flexible loops, are in (1) the positioning/tilt of the TM11 and TM12 helices, which serve as the dimerization interface in the full dimeric NKCC1 transporter, and (2) in the partial unfolding of the bend between TM4 and TM5 in the hNKCC1, which triggers a shift in the position of the two helices (Figure S8B). The observed differences propagate into more subtle structural changes in the inner core of the transporter, which in turn are reflected in the drug and/or ion binding. Specifically, the different tilting of the TM10 helix leads to change in the position of the hydrophobic pocket (F416, L603, F607 in zNKCC1; F495, L678, F682 in hNKCC1; Figure S8C), which, in turn, allows the aliphatic tail of BUM to adopt a different conformation. This distinct interaction is mirrored in the difference in the binding free energy contributions of these residues in the alanine scanning analysis (Tables S3 and S4). The TM10 tilting also influences the position of the Y611/Y686 residue in zNKCC1 and hNKCC1, respectively. While in hNKCC1 Y686 forms a stable hydrogen bond with BUM (occupancy > 90%), in zNKCC1 the Y611 interaction is weaker (H-bond occupancy ~50%). In addition, the subtle change in TM6 conformation leads to a perturbation of the bCl^−^ binding site (G421, L422, L423, A424 in zNKCC1; G500, I501, L502, A503 in hNKCC1), which also influences the BUM binding and also explains the difference in the bCl^−^ behavior between zNKCC1 and hNKCC1 orthologs (Figures S4 and S5).

The difference in the Na^+^ binding ability can, instead, be associated to the change in the conformation of TM8 (Figure S8B). Namely, residues S538 and S539 in zNKCC1 are too far from the Na^+^ binding site, as compared to the S613 and S614 in hNKCC1 (Figure S8D). The subsequently weakened Na^+^ binding in zNKCC1 is thus more susceptible to be disrupted upon BUM binding, consistent with the larger Na^+^ mobility in the BUM/zNKCC1.

### 2.3. Binding Mode of Other Loop Diuretic Drugs

The binding mode predicted for BUM was used as a reference for that of the other NKCC1 inhibitors. Namely, the initial poses of the remaining inhibitors were generated by docking the inhibitors on the same zNKCC1 binding pocket, also considering the same ion occupancy, and the resulting poses were relaxed by MD simulations.

#### 2.3.1. Azosemide Binding Mode

**Figure 4:**
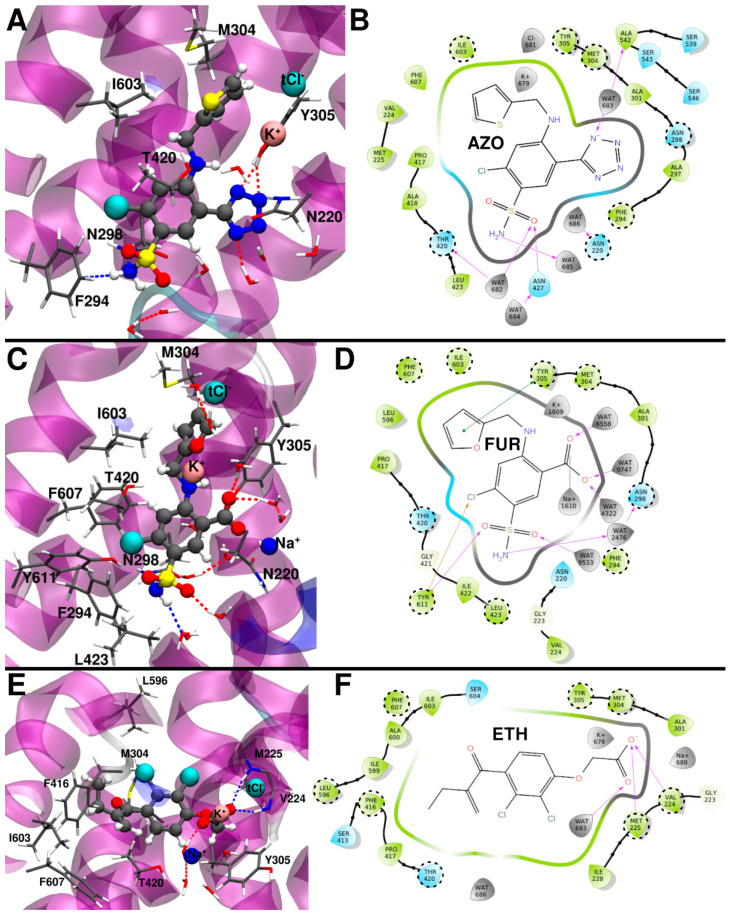
(**A**) Binding mode of azosemide (AZO) from the representative structure of the most populated cluster extracted from the molecular dynamics (MD) trajectory. (**B**) 2D schematic representation of the AZO/zNKCC1 interactions. (**C**) Binding mode of furosemide (FUR) from the representative structure of the most populated cluster extracted from the MD trajectory. (**D**) 2D schematic representation of the FUR/zNKCC1 interactions. (**E**) Binding mode of ethacrynic acid (ETH) from the representative structure of the most populated cluster from the MD trajectory. (**F**) 2D schematic representation of the ETH/zNKCC1 interactions. Polar/charged protein residues are colored blue, while the others are colored green. Waters and ions are colored gray. The most important protein residues, identified by alanine scanning analysis, are highlighted in dashed circles.

Interestingly, AZO is the only inhibitor not maintaining a stable binding pose in the holo zNKCC1 (i.e., with all three ions present in the structure). After ~350 ns of MD simulation the Na^+^ ion dissociated from zNKCC1 (Figure S9). The likely culprit for this dissociation is the N-heterocycle of the drug, which was initially positioned close to the Na^+^ ion. After Na^+^ dissociation, the MD simulation was extended to 800 ns to obtain an AZO/zNKCC1 trajectory of equal length to the other inhibitor/zNKCC1 complexes. In this part of the trajectory the other ions (K^+^ and tCl^−^) underwent a remodeling of their positions (Figure S9). This results in a similar ΔG_b_ of AZO (−20.6 ± 4.6 kcal/mol) towards zNKCC1 as compared to BUM (−21.7 ± 3.9 kcal/mol, Table 1). Nevertheless, the ions still mediate AZO binding with the K^+^ ion contributing to stabilize it (Table S5). In contrast to BUM, the AZO sulfamoyl group points towards the solvent exposed zNKCC1 exit channel and does not directly interact with the protein nor with the ions (Figure 4A,B). The alanine scanning analysis (Table S5) elucidates that the largest contribution to ΔG_b_ of AZO is Y305, which forms π-stacking and H-bond interactions to the drug’s thiophene and tetrazole rings, respectively. In addition, I603 and M302 “sandwich” the thiophene ring against the Y305. The drug’s sulfamoyl moiety instead establishes hydrophobic interactions with F294 and H-bonds to N298. Interestingly, the important residues identified by the alanine scanning analysis include, apart from M304, also F294, another residue pinpointed in mutagenesis studies to be important for the binding of loop diuretic inhibitors [25].

#### 2.3.2. Furosemide Binding Mode

Like BUM, FUR remained stably bound to holo zNKCC1 retaining all three ions (Figure S10). In this binding pose, K^+^ was partially coordinated by the drug (Figure 4C and Figure S10). Similar to the BUM/zNKCC1 complex, the Na^+^ ion underwent an initial repositioning before reaching a conformation stabilized by its interaction with the FUR carboxylic group. Both Na^+^ and K^+^ contributed significantly to the stabilization of the drug, while Cl^−^ again moderately destabilized it. Although FUR shares with BUM the sulfamoyl and carboxyl groups, these moieties exhibited different orientations (Figure 3A and Figure 4C) with the first pointing towards the exit channel and being partially solvent exposed, and the latter being oriented towards the Na^+^ ion. FUR shares with AZO a Cl substituent, which exhibited similar orientation in the two cases.

FUR has a markedly higher ΔG_b_ (ΔG_b_ = −16.9 ± 5.9 kcal/mol) than AZO and BUM. The alanine scanning analysis (Table S6), pinpointed F607, which π-stacks to the drug’s central ring, as the key binding residue. M304, I603 along with Y503 form the furan binding pocket. Y305 H-bonds and π-stacks with the carboxylic group and the furan ring of the drug. While Y611 H-bonds with its sulfamoyl group oxygen and the Cl atom. Finally, N220 and N298 establish water-mediated interactions with the FUR sulfamoyl group. Once more the M304 and F294 residues exert a key contribution to FUR binding, in agreement with experimental findings [25].

#### 2.3.3. Ethacrynic Acid Binding Mode

The last investigated drug was ETH, which exhibits a distinct scaffold from the other inhibitors due to its smaller size and the lack of the sulfamoyl moiety. The docking simulations resulted in two energetically similar, yet distinct, binding poses. These were both relaxed via 500 ns long MD simulations. After calculating the binding enthalpy (ΔH_b_), the most energetically favorable pose (Pose 1 in Figure S11) was selected and subjected to further investigation.

In this pose, ETH remained stable along the MD simulation exhibiting the carboxylic group primarily oriented towards K^+^ ion (Figure 4E), while the Na^+^ ion was quite mobile and moved closer to the zNKCC1 exit channel (Figure 4E and Figure S12). During the MD simulation the ETH aliphatic chain occupied a similar position as BUM, while its two Cl substituents were oriented towards the inner side of the zNKCC1 channel, not interfering/interacting with any of the two NKCC1 Cl^−^ binding sites. The ETH ΔG_b_ (−13.1 ± 5.0 kcal/mol) is the worst among all inhibitors studied. Based on the alanine scanning analysis (Table S7), the largest contributions to ETH ΔG_b_ come from the zNKCC1 hydrophobic residues: Y305, L596, V224, M304, F416, I603, F607, T420. Additionally in this case the Na^+^ and K^+^ ions favorably contribute to the ΔG_b_, while the tCl^−^ ion has a small negative impact. Consistent with the above detailed results for the other inhibitors, M304 is again among the key residues stabilizing ETH binding.

## 3. Discussion

Our detailed atomic-level investigation of the binding of distinct loop diuretics to the inward-facing conformation of NKCC1 supplies precious information about their putative binding poses. Overall, our results are in-line with the experimental data showing that BUM (IC_50_ ~1.0 μM) and AZO (IC_50_ ~0.2 µM) are more potent NKCC1 inhibitors than FUR (IC_50_ ~5–6 μM); and that ETH (IC_50_ ~1.6–3.0 mM) is the least efficient inhibitor [23]. While the comparatively very low IC_50_ of AZO cannot be explained by our simulation, it is likely that its higher potency is related to its favorable transport properties, tissue distribution and ability to more easily cross the blood–brain barrier due to the lack of the negatively charged carboxylic group, which is present in the other drugs [23]. Among the other common traits of the binding poses we list the role of monovalent ions in mediating and stabilizing the drug binding and the out competition of the most solvent exposed Cl^−^ ion (bCl^−^) by all inhibitors. Yet, while the BUM carboxylic group occupies the site, which before its dissociation coordinates the “bottom” bCl^−^ ion, none of the remaining inhibitors filled this site, highlighting a possible trait for drug-optimization campaigns.

Remarkably, the BUM binding mode is mostly conserved in both the z and hNKCC1 orthologs, although small differences are caused by subtle changes in the relative positioning of the transmembrane helices (Figure S8). In addition, the ions are more mobile in the apo hNKCC1 compared to the zNKCC1 (Figures S4 and S5). Specifically, the bCl^−^ is more mobile in hNKCC1 and can undergo multiple partial unbindings, which can be explained by a small remodeling of its binding site between zNKCC1 and hNKCC1 (Figure S8). Due to the cooperativity/interdependency of the ions transport [40], the mobility of bCl^−^ can subsequently influence the behavior of the remaining ions. In contrast, the Na^+^ is more stably bound in the BUM/hNKCC1 structure than in BUM/zNKCC1 thanks to a subtle repositioning of the TM8 helix, which distorts the Na^+^ binding site in zNKCC1. The observed differences may be in part due to the fact that the ion-bound hNKCC1 model was built on the basis of the positions occupied in the zNKCC1 model. Nonetheless, in support of our results, it should be noted that the ions behave differently in the distinct computational studies performed so far on NKCC1 [40,41]. Thus, the different ion behavior between distinct NKCC1 isoforms warrants further investigation.

Notably, the methionine residue (M304 in zNKCC1, M382 in hNKCC1), suggested to be key for BUM binding in mutagenesis studies [25], was also identified by our simulation as the key residue for the binding of all inhibitors. This supports the binding poses of the loop diuretics proposed here. Additionally, M304 has been suggested to act as a gate-residue, which controls the access of ions from the extracellular side to the inner NKCC1 cavity [25]. By engaging hydrophobic interactions with M304, the inhibitors can restrict its conformational plasticity, disrupt the gating, and stop the in-flow of ions in addition to sterically blocking the inner cavity of NKCC1.

Our simulations were performed on the inward-facing structure of NKCC1. Indeed, experimental evidence shows that BUM can be imported inside the cells by the Monocarboxylate Transporter 6 (MCT6) [26] and, in turn, exported from cells by three transporters: Multidrug Resistance Protein 4 (MRP4), Organic Anion Transporter 3 (OAT3) and Organic Anion Transporting Polypeptide 2 (OATP2) [28]. Indeed, when the latter are inhibited, the available in cell BUM concentration increases [29]. This evidence suggests that BUM can bind NKCC1 from the intracellular side. Nevertheless, it is also possible that the loop diuretic inhibitors may exert their biological function by binding at the extracellular side to the NKCC1, when in the outward facing conformation. A recent work, published while we were finalizing this manuscript, introduced mutations to block the NKCC1 in the outward-facing conformation and trap BUM inside, showing that this may be a possible alternative [42]. A qualitative analysis of this latter BUM binding mode to the outward-facing NKCC1 (Figure S13, Table S8) shows that BUM in this binding pose exhibits a binding free energy comparable to that obtained in this study. In addition, while we were revising the manuscript, a new structure of the human NKCC1 was released showing yet another possible binding site of the loop diuretics located in the intracellularly-exposed C-terminal domain [43]. This new finding supports the proposition that BUM can cross the cell membrane and bind to NKCC1 from the cytosolic side and suggests that the loop diuretic binding modes/mechanism are perhaps more intricate than originally envisaged.

In summary, here we supply an atomic-level characterization of a possible binding mode of BUM and related loop diuretic inhibitors to the inward-facing conformation of the human and zebrafish NKCC1. All drugs retain a similar binding mode occupying a cytosolic cavity and establishing interactions with the residues involved in ion gating, thus supplying a rational to their inhibition mechanism. Consistent with experimental evidence, our study reveals that K^+^/Na^+^ ions are key for the binding of the loop diuretics, while of the two Cl^−^ ions, one remained bound and has a negative impact on drug binding, while the other was out competed by the drugs. Despite their similar general binding pose, the inhibitors differ in their specific NKCC1 and/or ion interactions. As well, differences are also observed in the binding free energy (ΔG_b_) and in the Na^+^ behavior between zNKCC1/BUM and hNKCC1/BUM, pinpointing a distinct ion mobility/plasticity in the two orthologs. This has been consistently observed in other computational studies of the NKCC1 ion transport mechanism [40,41]. Our findings provide a paradigmatic example of the pivotal role of monovalent metal ions dynamical adaptability to mediate the binding of drugs to metal ion transporters. Briefly, this work highlights the importance of accounting for metal ion dynamical effects to accomplish their repositioning and/or dissociation during drug binding.

Taken together our results provide atomic-level insights on the inhibition mechanism of NKCC1 to be used in forthcoming drug discovery campaigns aimed at tackling human diseases linked to a deregulated Cl^−^ homeostasis.

## 4. Materials and Methods

### 4.1. Binding Site Location and Docking

In order to identify the possible NKCC1 binding sites of the loop diuretics, we used the FTMap site detection algorithm [44] on the first and more complete structure of NKCC1 solved by cryo-EM studies. This was the zebrafish NKCC1 (zNKCC1) structure (PDB id: 6NPL), which accounts for the whole dimeric transmembrane portion and the cytosolic C-term domain, and for the position of most of the transported ions (K^+^ and Cl^−^) [35]. Two possible binding hot-spots were detected: one at the interface of the two transmembrane domains, which is normally occupied by lipid molecules [35], and second one inside the transmembrane domain in the vicinity of the ion binding sites. Since BUM is known to compete with Cl^−^ ions for its binding [3,18], we used this second druggable pocket to dock BUM into the zNKCC1 structure using the SWISSDOCK webserver [45]. The BUM/NKCC1 complex was then relaxed for 500 ns of molecular dynamics (MD), and its binding pose was refined with metadynamics (MTD) simulations. The details of these simulations are reported below. The refined binding pose of BUM was then used to dock the other NKCC1 inhibitors: azosemide (AZO), furosemide (FUR) and ethacrynic acid (ETH). Protonation states of the ligands were generated using the Ligand Preparation protocol and the docking was performed using Glide XP from Schrödinger Suite [46]. We considered the top five docking poses per each ligand. The AZO and FUR docking resulted in only one distinct binding pose, while the docking of ETH resulted in two distinct binding poses (Figure S11). The best ranked binding pose of AZO and FUR and the two binding poses of ETH were then considered in subsequent simulations.

### 4.2. Model Building

Two models of NKCC1 were built in this study. (I) The monomeric zNKCC1 model of the transmembrane domain was built from the cryo-EM structure (PDB id: 6NPL) as in our previous study [35]. (II) The model of human NKCC1 (hNKCC1) transmembrane portion was built based on the cryo-EM structure (PDB id: 7D10) of the human NKCC1 dimer [36]. Preparation of the second model followed the same procedure detailed in our previous study [40]. Since the latter structure lacks the ions, their positions were assigned on the basis of the zNKCC1 model by superimposing the two structures. Both models were inserted into a pre-equilibrated 1-palmitoil-2-oleoyl-sn-glycero-3-phosphocholine (POPC) membrane bilayer using CHARMM-GUI [47,48]. For both models the size of the membrane was 110 Å × 110 Å, which contained 158 and 152 lipids in upper and lower leaflet, respectively. After the addition of a 22.5 Å layer of water in the z direction and 150 mM concentration of NaCl, the total number of atoms was ~140 k. The systems were described using Amber FF14SB [49] and Lipid17 [50] force fields (FF) for the protein and POPC lipids, respectively; and were solvated using the TIP3P water model [51]. Joung and Chetham parameters were used for all the Na^+^, Cl^−^ and K^+^ ions [52]. The inhibitors were described using gaff FF [53] with RESP charges obtained using the REDD webserver [54]. We initially used the zNKCC1 model as the primary model for the study of the binding of different NKCC1 inhibitors. Then the hNKCC1 model, was used to verify the validity/relevance of the binding pose predicted for BUM on zNKCC1.

### 4.3. Molecular Dynamics Simulation Details

Molecular dynamics (MD) simulations were performed using GROMACS 2020 program [55,56]. The systems were equilibrated in 8 steps with an MD simulations length of 0.5 ns, 0.25 ns, 0.5 ns, 0.5 ns, 0.5 ns, 0.5 ns, 0.5 ns, 2.0 ns and progressively decreasing the restraints on the protein (5000 kJ/mol·nm, 4000 kJ/mol·nm, 3000 kJ/mol·nm, 2000 kJ/mol·nm, 1000 kJ/mol·nm, 500 kJ/mol·nm, 250 kJ/mol·nm, 50 kJ/mol·nm, 0 kJ/mol·nm) and the membrane (2000 kJ/mol·nm, 2000 kJ/mol·nm, 1000 kJ/mol·nm, 500 kJ/mol·nm, 250 kJ/mol·nm, 100 kJ/mol·nm, 50 kJ/mol·nm, 0 kJ/mol·nm, 0 kJ/mol·nm). Restraints on Cα atoms with 50 kJ/mol·nm were applied in the last step. The Cα restraints on the transmembrane part of NKCC1 were kept for the first 30 ns of MD simulations to allow for membrane equilibration. The MD simulations were then extended to 500 ns without restraints. The BUM binding pose predicted by docking was relaxed by 500 ns-long MD simulation before running metadynamics (MTD) simulation (detailed below) and the most stable minimum obtained from MTD was further relaxed by a 500 ns-long unbiased and unrestrained MD simulation. The same pose was then also used to infer the binding pose of BUM bound to the hNKCC1 ortholog.

The binding poses of the other loop diuretics (AZO, FUR and EHT) obtained in docking studies on zNKCC1 were then again relaxed by performing 500 ns long MD simulation with the exception of the zNKCC1/AZO system, whose simulation was extended to 800 ns. The most representative protein–ligand conformations were extracted by hierarchical agglomerative clustering analysis using cpptraj from AmberTools [57] for visualization.

### 4.4. Metadynamics Simulation Details

In order to refine the initial docked position of BUM in complex with zNKCC1, we performed MTD with the GROMACS 2020 coupled with PLUMED 2.7 plugin [58]. For this purpose, we utilized the well-tempered (WT) volumetric-based MTD simulations [59], which we previously applied to study the ion transport process of NKCC1 [40]. This approach employs spherical coordinates of the ligand (ρ, θ, φ) as CVs for the WT-MTD simulations. The ρ CV represents the distance of the ligand from the center of the coordinate system, which here corresponds to the center of mass of the core NKCC1 helices (H1, H2, H3, H6, H7, H8, H10). The θ and φ CVs define angles accounting for the orientation of the ligand within a sphere of radius ρ. The sampling space is limited by restraining the ρ coordinate at 20 Å. This corresponds to the distance where the ligand reaches the protein surface/channel exit. The restraint at this ρ value does not allow the full dissociation of the ligand. This prevents attaining reliable binding free energy of the ligand. Nonetheless, this approach accelerates the sampling of drug binding/unbinding events and enables us to explore different ligand binding poses within the NKCC1 and to assess the most energetically favorable one. The parameters used for WT-MTD simulations were as follows: deposition time = 5 ps, hill height = 2.0 kJ/mol, σ_rho_ = 0.5 Å, σ_theta_ = π/16, σ_phi_ = π/8, and bias factor = 10, following a previously applied protocol [40]. Each MTD simulation was run for 400 ns.

For analysis purposes the free energy information was reweighted onto a 2D surface using the distance from the center, ρ, as CV1, and the number of contacts of the ligand with protein inner cavity (residues 216–225, 297, 298, 300–305, 364, 413, 414, 416–424, 427, 454, 532, 535, 536, 542, 543, 546, 596, 600, 602–608, 611; Figure S14), as CV2. The number of contacts was expressed as a coordination number between ligand non-hydrogen atoms and the selected protein non-hydrogen atoms using the formula: $CN=\sum_{i\in A}\sum_{i\in B}\frac{1-{\left(\frac{r_{ij}}{r_{0}}\right)}^{6}}{1-{\left(\frac{r_{ij}}{r_{0}}\right)}^{12}}$ where *A* is the set of non-hydrogen atoms of the ligand, *B* the set of non-hydrogen atoms of the protein and the *r*_0_ is a threshold to define a formed contact, *r*_0_ = 4.5 Å. Furthermore, the identification of minima was performed using metadynminer3d [60] on the 3D free energy surface of the original 3 CVs (ρ, θ, φ) in order to avoid possible artifacts resulting from the reweighting procedure.

To establish the effect of the NKCC1-bound ions on the BUM binding, two separate BUM-refining MTD simulations were run: one with the zNKCC1 in the apo state and the other with the K^+^, Na^+^ and one Cl^−^ bound to the zNKCC1. In the ion-bound MTD simulation, additional wall restraints were placed on distances of the ions from the centers of their respective binding sites. These walls were placed at a distance value of 2.0 Å with a force constant of 250 kJ/mol·nm in order to prevent ion dissociation due to displacement by BUM, while still enabling their remodeling when BUM explores the different binding modes within the NKCC1 cavity. In the follow-up unbiased MD simulations, the restraints on the ions were removed in order to allow their remodeling in response to BUM binding.

### 4.5. MM/PBSA Binding Free Energy Calculations and Alanine Scanning Analysis

Molecular mechanics (MM) with Poisson–Boltzmann (PB) and surface area (SA) solvation (MM/PBSA) method, as implemented in MMPBSA.py [61] from AmberTools, was used to calculate the binding free energy of the bound inhibitors. In all cases, 50 frames from the equilibrated part of the trajectory were used for the free energy calculation. Since NKCC1 is membrane-embedded protein, the membrane correction to PBSA was used [62]. Slab-like implicit membrane with a spline-fitted dielectric constant depth profile was used (membraneopt = 3) with the pore searching algorithm turned on (poretype = 1). The thickness of the membrane was assumed to be 40 Å, which corresponds to the used POPC membrane. The external dielectric constant was 80, while the internal one was 20 and the membrane one was 7; these values were adopted from [62]. The ionic strength was set to account for the 150 mM ion concentration used in the MD simulations. The NKCC1-bound ions, as well as 8 water molecules nearest to the ions, were explicitly included in the MM/PBSA calculation as part of the receptor. The entropy contribution to the binding free energy was calculated by using the interaction entropy scheme [63].

We also estimated the role of different NKCC1 residues in ligand binding by performing in silico alanine scanning analysis. For each of the four drugs, we selected the residue within 5 Å of the drug for the alanine mutation and subsequent re-calculation of the MM/PBSA binding free energy, using the original trajectory, to assess their contribution to the drug binding. Thus, this analysis does not account for the possible structural effects of the mutations, but it allows us to estimate the energetic contribution of each residue. Alanine, glycine, and proline residues were excluded from the analysis. Four experimentally identified residues (I293, F294, V300, M304) were also included in the analysis [25]. The entropy contribution was not calculated for the alanine scanning analysis.

## Figures and Tables

**Table 1:** Binding free energies (ΔG_b_, kcal/mol)) of different NKCC1 inhibitors as obtained by Molecular Mechanics Poisson Boltzmann Surface Area (MM/PBSA). Enthalpic (ΔH_b_) and entropic (−TΔS) contribution to the ΔG_b_ are provided. Standard deviations of all values are reported.

	ΔH_b_ [kcal/mol]	−TΔS [kcal/mol]	ΔG_b_ [kcal/mol]
Bumetanide zNKCC1	−27.8 ± 3.1	6.1 ± 2.3	−21.7 ± 3.9
Bumetanide hNKCC1	−32.6 ± 3.4	6.2 ± 3.3	−26.4 ± 4.7
Azosemide	−27.6 ± 3.3	7.0 ± 3.2	−20.6 ± 4.6
Furosemide	−24.8 ± 4.3	7.9 ± 4.0	−16.9 ± 5.9
Ethacrynic acid	−22.1 ± 3.6	9.0 ± 3.4	−13.1 ± 5.0

## Data Availability

The data presented in this study are not publicly available due to the large size of the MD trajectory data. Yet they are available on request to the corresponding author.

## Supplementary Materials

The following supporting information can be downloaded at: https://www.mdpi.com/article/10.3390/ijms232315439/s1. References [42,64,65] are cited in the supplementary materials.
