# Supplementary material for: Role of Monovalent Ions in the NKCC1 Inhibition Mechanism Revealed through Molecular Simulations
Source: Int J Mol Sci. 2022 Dec 6;23(23):15439. doi: 10.3390/ijms232315439 (PMC9741434; doi:10.3390/ijms232315439)
Supplement: Supplementary file 1 [file ijms-23-15439-s001.zip › ijms-1968982-SI.pdf]

Supporting Information

Role of monovalent ions in the NKCC1 inhibition mechanism revealed through molecular simulations

Pavel Janoš❁, Alessandra Magistrato❁

❁ CNR-IOM c/o International School for Advanced studies (SISSA/ISAS), via Bonomea 265, 34136, Trieste, Italy

Table of Contents

Metadynamics refinement of the bumetadine binding pose	page 2
Bumetanide binding to zebrafish NKCC1	page 6
Ion behavior in zebrafish and human NKCC1 orthologs	page 8
Bumetanide binding to human NKCC1	page 10
zNKCC1 and hNKCC1 comparison	page 12
Azosemide binding to zebrafish NKCC1	page 14
Furosemide binding to zebrafish NKCC1	page 16
Docked poses of azosemide, furosemide and ethacrynic acid	page 18
Ethacrynic acid binding to zebrafish NKCC1	page 19
Bumetanide binding to outward-facing hNKCC1	page 21
Inner cavity definition	page 22

Metadynamics refinement of the bumetadine binding pose

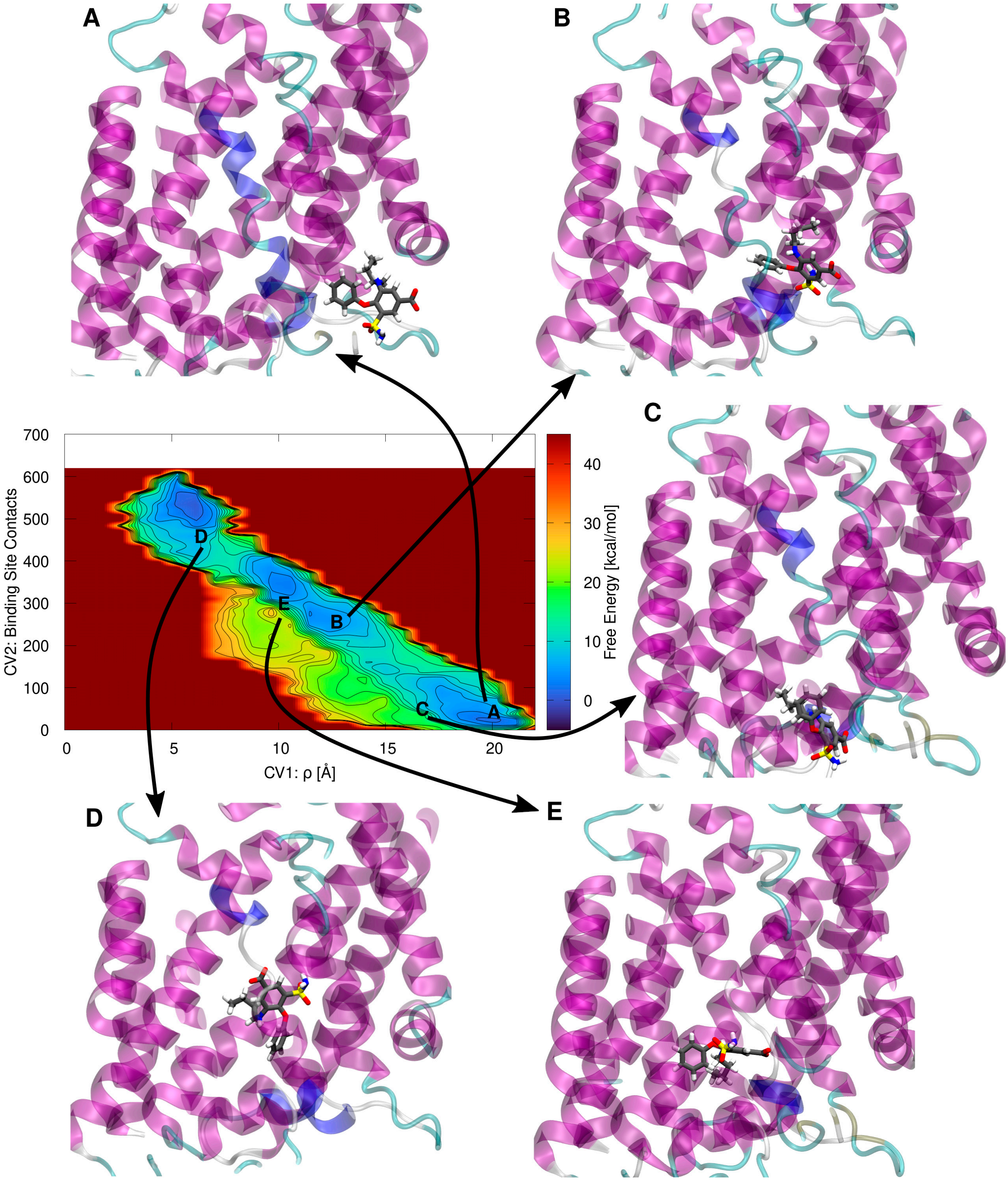

Figure S1. Graphical representation of the minima identified in the volume-based metadynamics simulation of the zebrafish NKCC1 model for bumetanide (BUM) in the absence of ions bound to the transporter. NKCC1 is depicted as magenta new cartoons with loops depicted in cyan. The BUM is shown in licorice and colored by atom name. Free energy surface of the BUM (un)binding is plotted as a function of the ligand center of mass with respect to the center of mass of the protein CV1 ρ(Å) and the number of BUM contacts with the active site residues (CV2). Free energy is reported in color ranging from dark blue to dark red. Contour lines are reported every 2.0 kcal/mol. Minima listed in Table S1.

Table S1. Minima identified in the bumetanide volume-based metadynamics simulation in the absence of bound ions within the zebrafish NKCC1 model.

Minima	ρ [A]	θ [rad]	φ [rad]	Relative free energy [kcal/mol]	Binding site contacts
A	19.37	2.14	0.45	-44.9	44
B	12.40	2.24	0.65	-43.6	256
C	16.40	2.94	0.35	-32.6	50
D	6.07	2.29	1.65	-31.3	460
E	9.40	2.94	1.35	-27.7	300

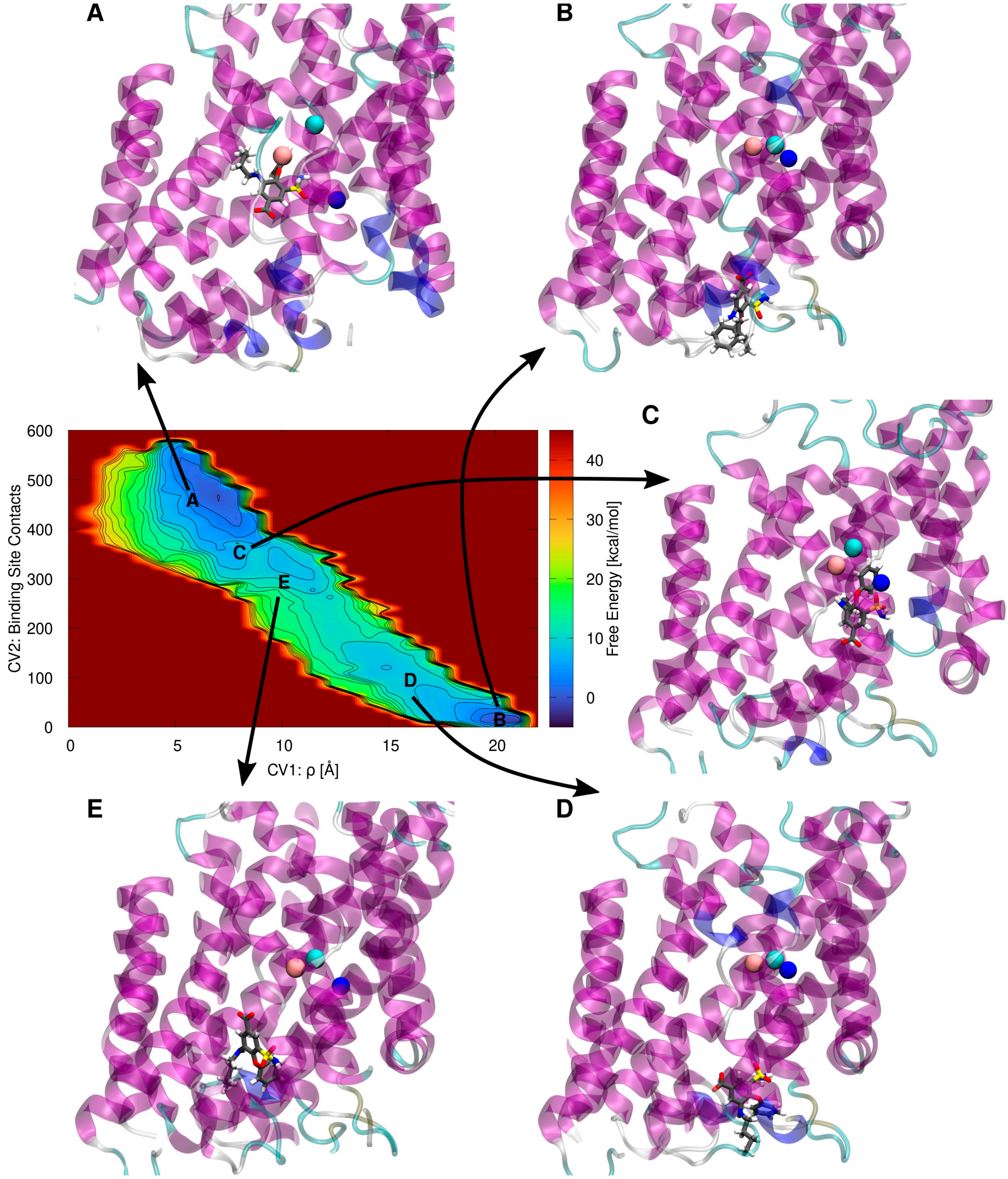

Figure S2. Graphical representation of the minima identified in the volume-based MTD simulation of the zebrafish NKCC1 model for bumetanide (BUM) in the presence of the monovalent ions bound to the transporter. NKCC1 is depicted as magenta new cartoons with loops colored in cyan. The Cl^{-}, Na^{+}, and K^{+} ions are shown as cyan, blue, and orange van der Waals spheres, respectively. The BUM is shown in licorice and colored by atom name. Free energy surface of the BUM (un)binding is plotted as a function of the ligand center of mass with respect to the center of mass of the protein CV1 ρ(Å) and the number of BUM contacts with the active site residues (CV2). Free energy is reported in color ranging from dark blue to dark red. Contour lines are reported every 2.0 kcal/mol. Minima listed in Table S2.

Table S2. Minima identified in the BUM volume-based metadynamics smulation in the presence of the K^+, Na^+ and tCl^- ions in the zebrafish NKCC1 model. Minima shown in Figure S4 above.

Minima	ρ [A]	θ [rad]	φ [rad]	Relative free energy [kcal/mol]	Binding site contacts
A	4.97	0.95	1.05	-44.4	460
B	19.87	2.54	1.35	-40.9	15
C	7.74	1.65	0.85	-38.4	353
D	15.71	2.49	1.25	-36.9	96
E	9.82	2.59	1.94	-31.2	293

Bumetanide binding to zebrafish NKCC1

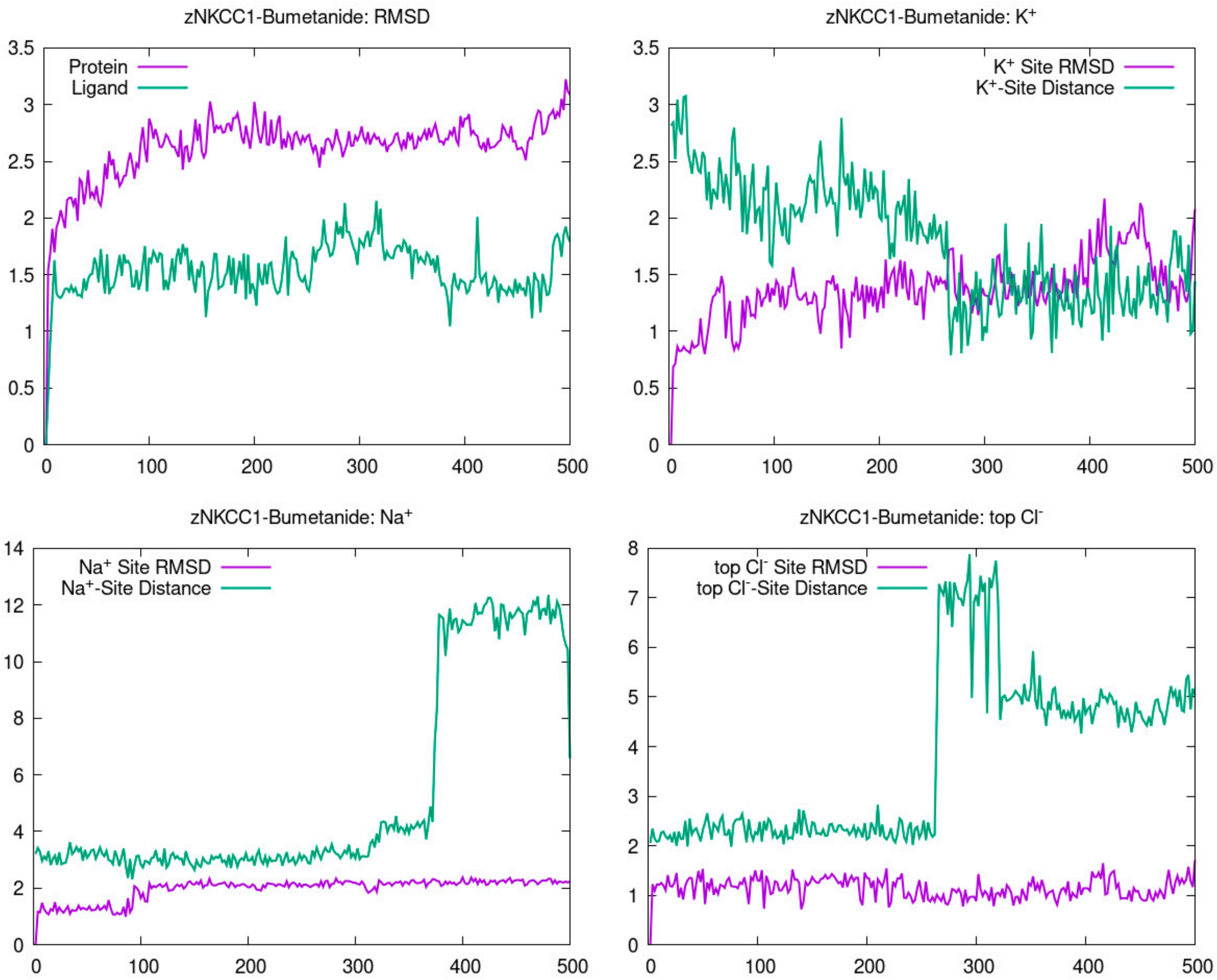

Figure S3. Root mean square deviation (RMSD, Å) of the bumetanide/zebrafish NKCC1 complex for the protein (violet line, top left) and drug (green line, top left). RMSD of the ion binding sites with respect to the first frame of the simulation (violet lines) and distance of the ion with respect to the center its ion binding site (green lines) vs simulation time (ns) for K^+ (top right), Na^+ (bottom left), the top Cl^- (bottom right).

Table S3. Molecular Mechanics Poisson Boltzmann Surface Area (MM/PBSA) binding free energies (ΔG_b , kcal/mol) per residue as obtained by the alanine scanning of the zebrafish NKCC1/bumetanide complex. The relative binding free energies ($\Delta\Delta G_b$) are calculated as ΔG_b of mutant - ΔG_b of wild type. The most important residues (with $\Delta\Delta G_b$ > 0.6 kcal/mol) are highlighted in bold. Residues identified in mutagenesis studies as important for the bumetanide inhibition mechanism are marked with *. Values from the MM/PBSA calculation in the absence of one of the ions are also provided to evaluate the importance of the ions for the bumetanide binding.

	$\Delta G_{binding}$ (kcal/mol)	Std. Dev.	Std. Err. of Mean	$\Delta\Delta G_{binding}$ (kcal/mol)
WT	-27.80	3.09	0.62	0.00
L219A	-27.80	3.09	0.62	0.00
**N220A**	**-27.17**	3.02	0.60	**0.63**
I221A	-27.73	3.09	0.62	0.07
W222A	-27.80	3.09	0.62	0.00
V224A	-27.80	3.08	0.62	0.00
M225A	-27.77	3.08	0.62	0.04
T253A	-27.79	3.09	0.62	0.01
I293A*	-27.80	3.09	0.62	0.00
F294A*	-27.77	3.15	0.63	0.03
N298A	-27.52	3.26	0.65	0.28
V300A*	-27.67	3.10	0.62	0.14
**M304A***	**-26.83**	3.00	0.60	**0.97**
**Y305A**	**-26.66**	3.01	0.60	**1.14**
S413A	-27.80	3.09	0.62	0.00
**F416A**	**-26.35**	3.13	0.63	**1.45**
T420A	-27.38	3.20	0.64	0.42
I422A	-27.34	3.11	0.62	0.46
**L423A**	**-27.25**	3.07	0.61	**0.55**
T536A	-27.78	3.09	0.62	0.02
S539A	-27.59	3.02	0.60	0.21
S543A	-27.67	3.13	0.63	0.13
L596A	-27.79	3.09	0.62	0.01
I599A	-27.81	3.09	0.62	-0.01
**I603A**	**-25.92**	3.28	0.66	**1.88**
S604A	-27.75	2.97	0.59	0.05
**F607A**	**-23.48**	3.34	0.67	**4.32**
L608A	-27.79	3.09	0.62	0.01
Y611A	-27.55	3.04	0.61	0.25
Na^+	-26.12	2.96	0.59	1.69
K^+	-25.10	3.16	0.63	2.70
Cl^-	-28.60	3.09	0.62	-0.80

Ion behavior in zebrafish and human NKCC1 orthologs

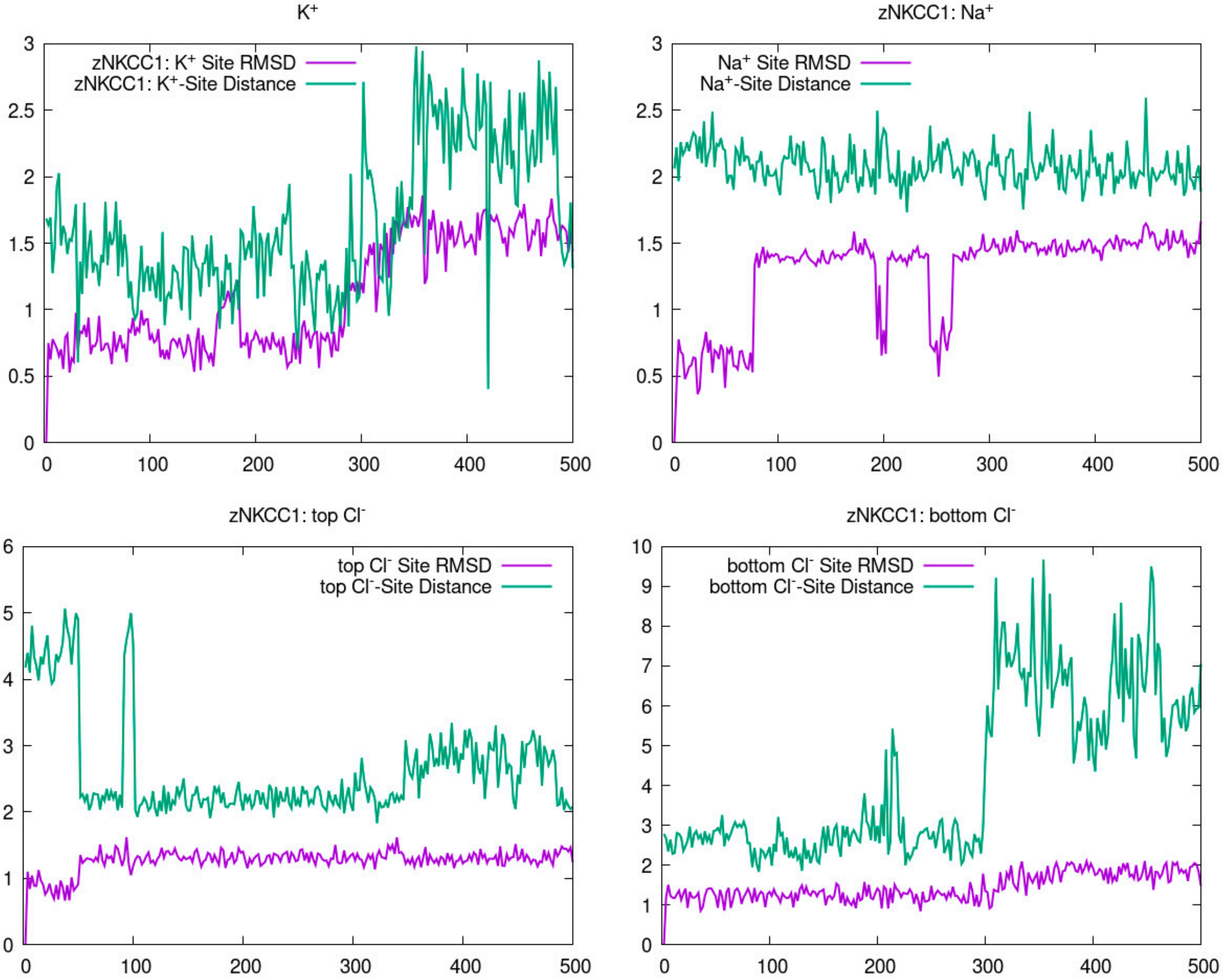

Figure S4. Ion behavior in the zebrafish NKCC1 model. Root mean square deviation (RMSD, Å) of the ions binding site calculated with respect to the first frame of the simulation (violet lines) and distance of the ion with respect to the center of its ion binding site (green lines) vs simulation time (ns) for K^+ (top left), Na^+ (top right), the top Cl^- (bottom left) and the bottom Cl^- ions (bottom right).

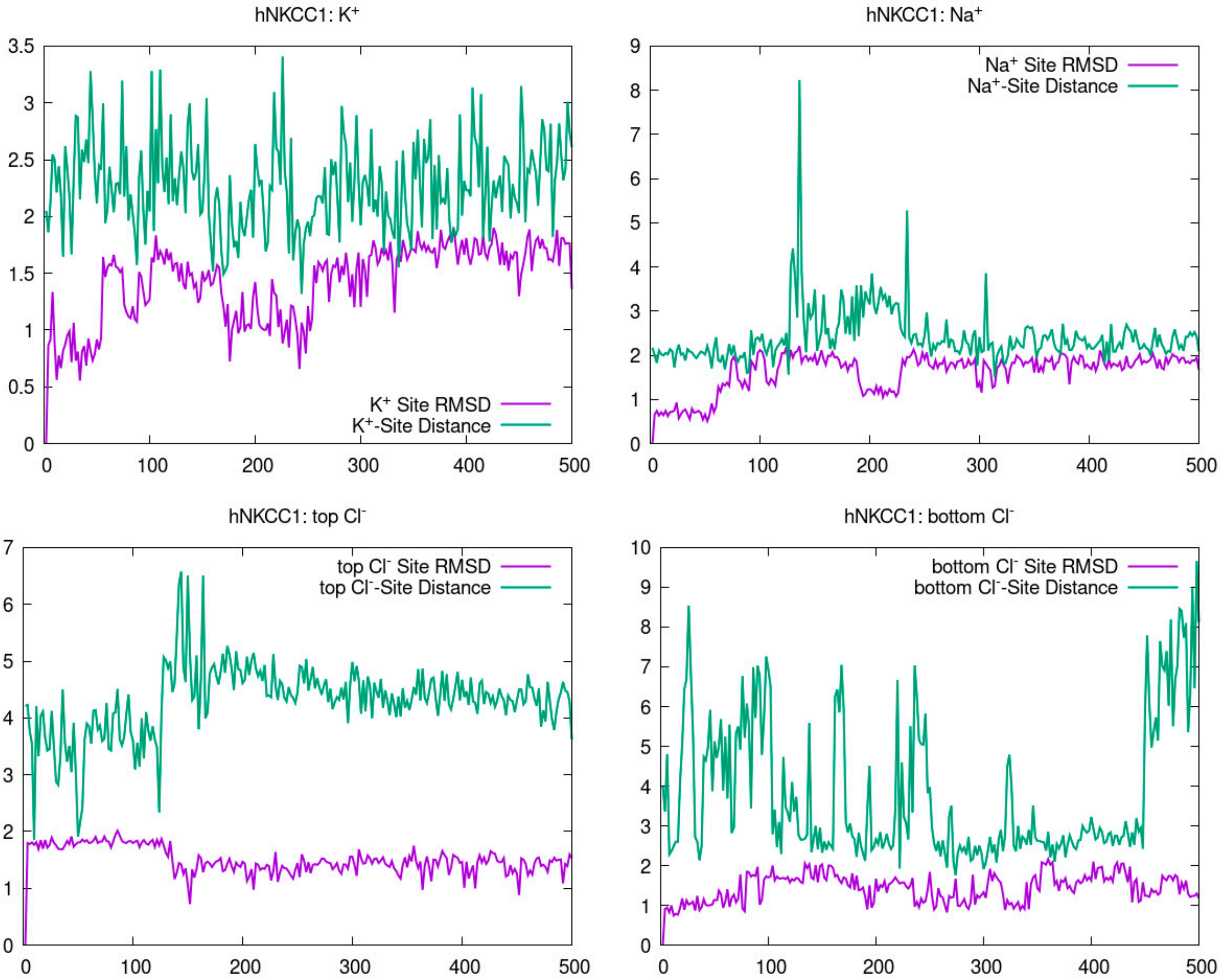

Figure S5. Ion behavior in the human NKCC1 model. Root mean square deviation (RMSD, Å) of the ions binding site calculated with respect to the first frame of the simulation (violet lines) and distance of the ion with respect to the center of its ion binding site (green lines) vs simulation time (ns) for K^+ (top left), Na^+ (top right), the top Cl^- (bottom left) and the bottom Cl^- ions (bottom right).

Bumetanide binding to human NKCC1

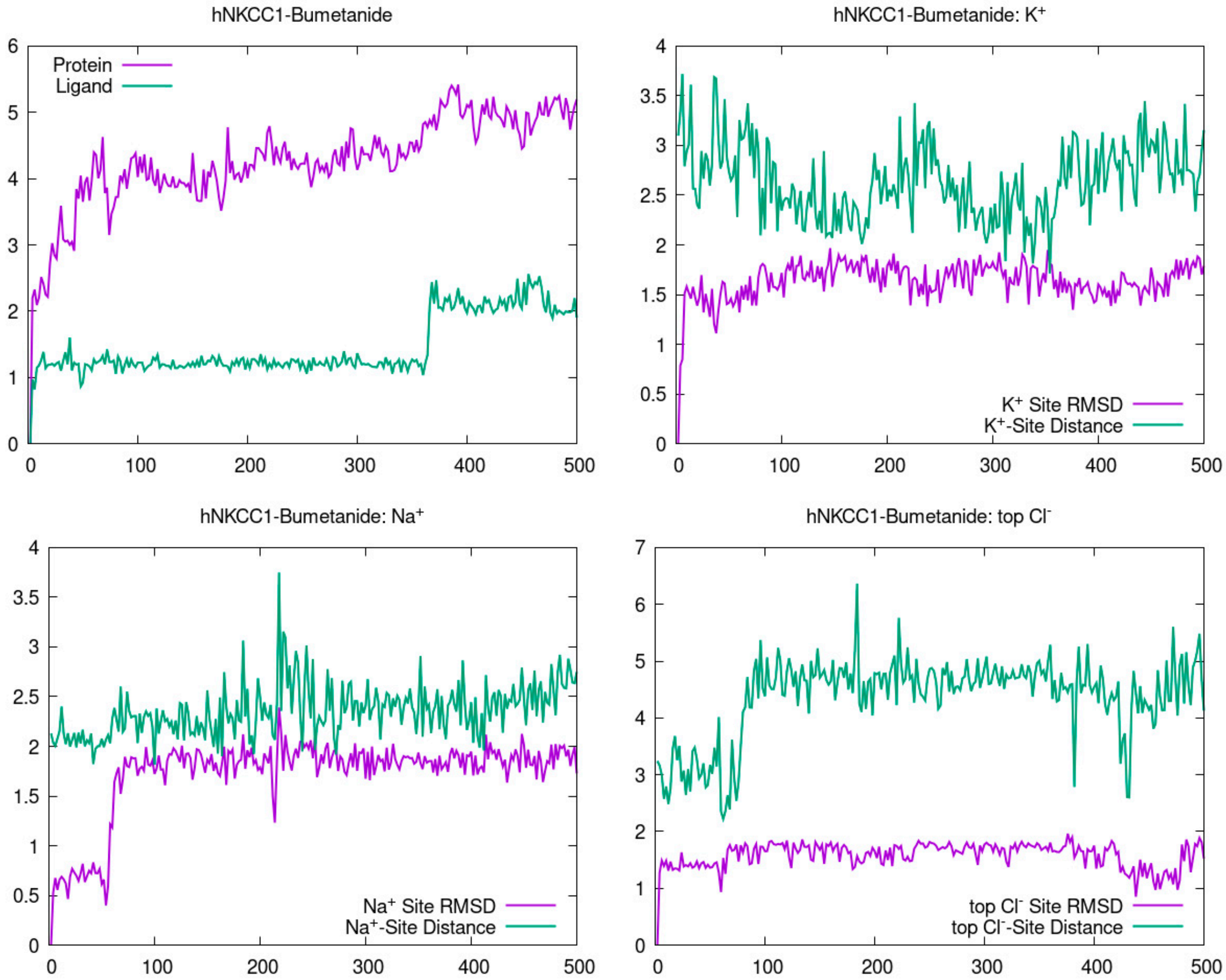

Figure S6. Root mean square deviation (RMSD, Å) of the bumetanide/human NKCC1 complex for the protein (violet line, top left) and drug (green line, top left). RMSD of the ion binding sites with respect to the first frame of the simulation (violet lines) and distance of the ion with respect to the center of its ion binding site (green lines) vs simulation time (ns) for K^+ (top right), Na^+ (bottom left), the top Cl^- (bottom right).

Table S4. Molecular Mechanics Poisson Boltzmann Surface Area (MM/PBSA) binding free energies (ΔG_b, kcal/mol) per residue as obtained by the alanine scanning of the human NKCC1/bumetanide complex. The relative binding free energies ($\Delta\Delta G_b$) are calculated as ΔG_b of mutant - ΔG_b of wild type. The most important residues (with $\Delta\Delta G_b > 0.6$ kcal/mol) are highlighted in bold. Residues important pinpointed in mutagenesis studies as important for the bumetanide inhibition are marked with *. Values from the MM/PBSA calculation in the absence of one of the ions are also provided to evaluate the importance of the ions for the bumetanide binding.

	$\Delta G_{binding}$ (kcal/mol)	Std. Dev.	Std. Err. of Mean	$\Delta\Delta G_{binding}$ (kcal/mol)
WT	-32.63	3.37	0.67	0.00
L297A	-32.63	3.37	0.67	0.00
N298A	-32.49	3.32	0.66	0.14
I299A	-32.52	3.36	0.67	0.11
W300A	-32.60	3.37	0.67	0.03
V302A	-32.49	3.36	0.67	0.14
M303A	-32.49	3.35	0.67	0.14
V330A	-32.62	3.39	0.68	0.01
I371A*	-32.62	3.37	0.67	0.01
F372A*	-32.58	3.35	0.67	0.05
N376A	-32.55	3.33	0.67	0.08
V378A*	-32.61	3.37	0.67	0.02
**M382A***	**-30.95**	3.36	0.67	**1.68**
**Y383A**	**-29.23**	3.45	0.69	**3.40**
F494A	-32.62	3.37	0.67	0.01
**F495A**	**-30.58**	3.37	0.67	**2.05**
T499A	-32.16	3.05	0.61	0.47
I501A	-32.34	3.37	0.67	0.29
L502A	-32.13	3.34	0.67	0.50
S614A	-32.61	3.35	0.67	0.02
S618A	-32.57	3.38	0.68	0.06
S621A	-32.59	3.37	0.67	0.04
I674A	-32.60	3.37	0.67	0.03
I677A	-32.63	3.37	0.67	0.00
**I678A**	**-30.39**	3.48	0.70	**2.25**
S679A	-32.46	3.34	0.67	0.17
**F682A**	**-28.36**	3.37	0.67	**4.27**
L683A	-31.82	3.30	0.66	0.81
S685A	-32.63	3.35	0.67	0.00
**Y686A**	**-30.56**	2.98	0.60	**2.07**
I730A	-32.57	3.35	0.67	0.06
Na^+	-31.67	3.41	0.68	0.96
K^+	-29.98	3.48	0.70	2.65
Cl^-	-33.01	3.48	0.70	-0.38

zNKCC1 and hNKCC1 comparison

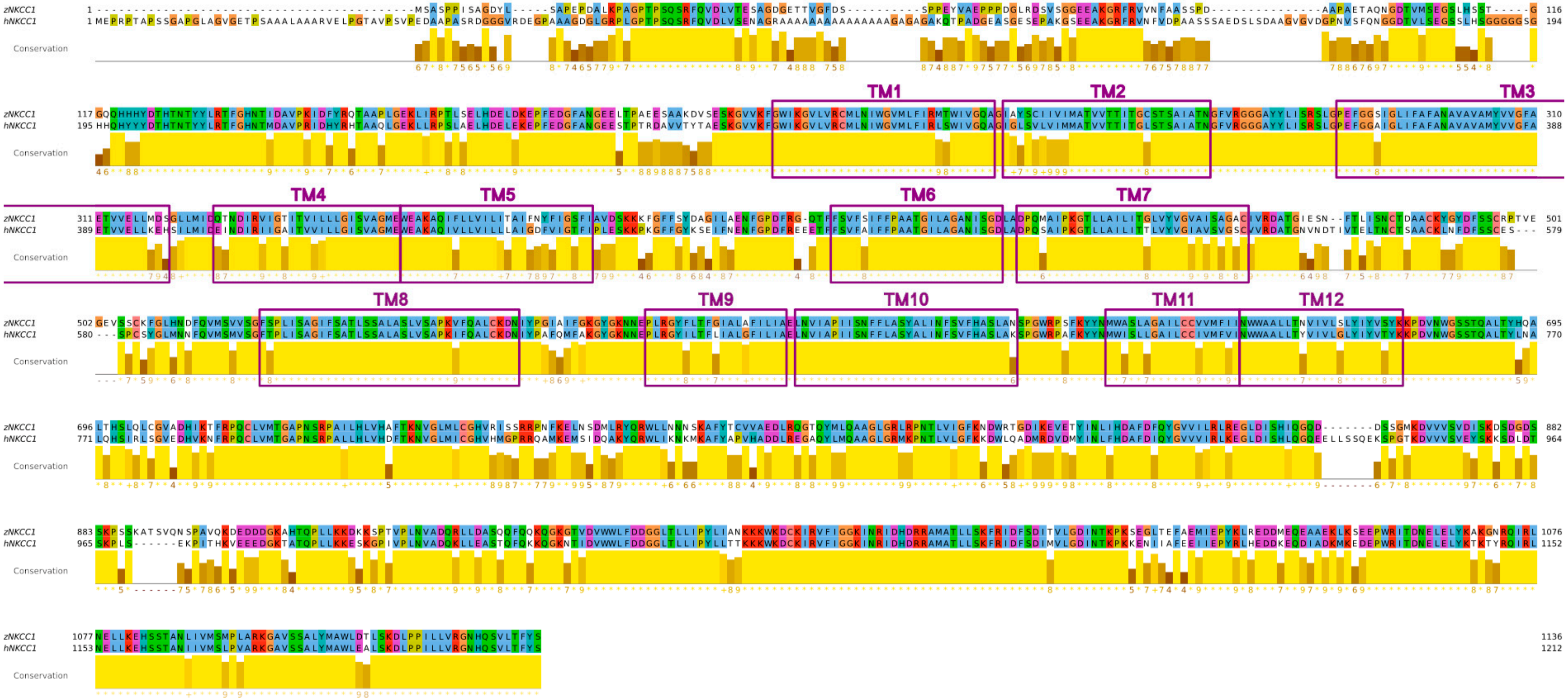

Figure S7. Sequence alignment of the zNKCC1 and hNKCC1 and the degree of the sequence conservation (going from brown to yellow for low to high conservation). The sequence alignment was performed by Clustal Omega [64] and visualized in Jalview [65]. Residues are color according to standard ClustalX coloring scheme: blue - hydrophobic, red - positive charge, magenta - negative charge, green - polar, pink - cysteins, orange - glycines, yellow - prolines, cyan - aromatic, white - unconserved.

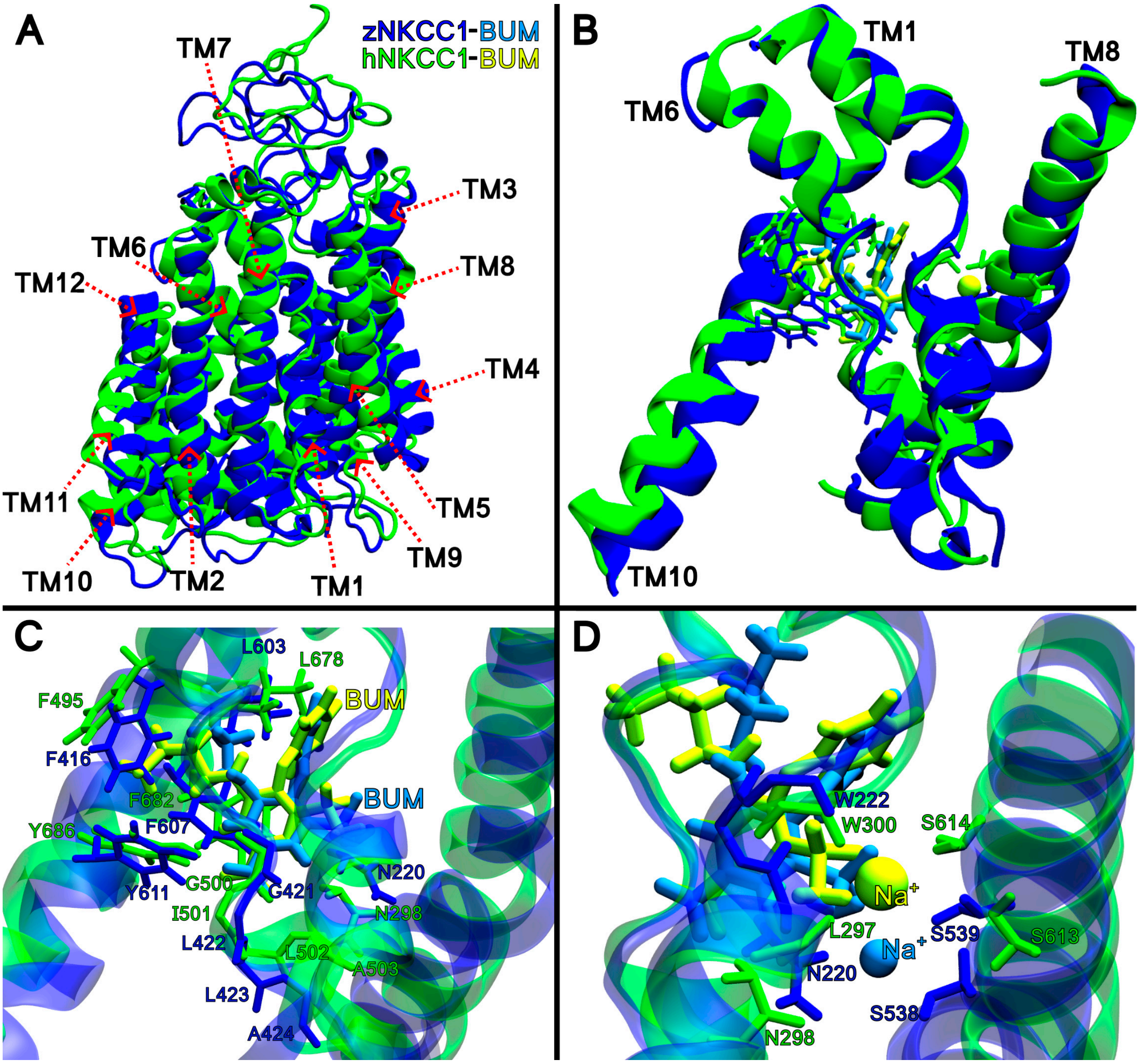

Figure S8. Structural comparison between the zNKCC1 and hNKCC1 orthologs with the BUM drug bound. (A) Overall view of the zNKCC1 and hNKCC1 with the transmembrane helices labeled as TM1-TM12. (B) Close-up view of the inner TM helices TM1, TM6, TM8 and TM10 that form the BUM and ions binding cavity. (C) Close-up view of the BUM binding site showing the subtle difference in the BUM binding pose in the zNKCC1 and hNKCC1 orthologs. (D) Close-up view of the Na^+ binding site showing the subtle structural difference leading to different behavior of the Na^+ ion between zNKCC1 and hNKCC1.

Azosemide binding to zebrafish NKCC1

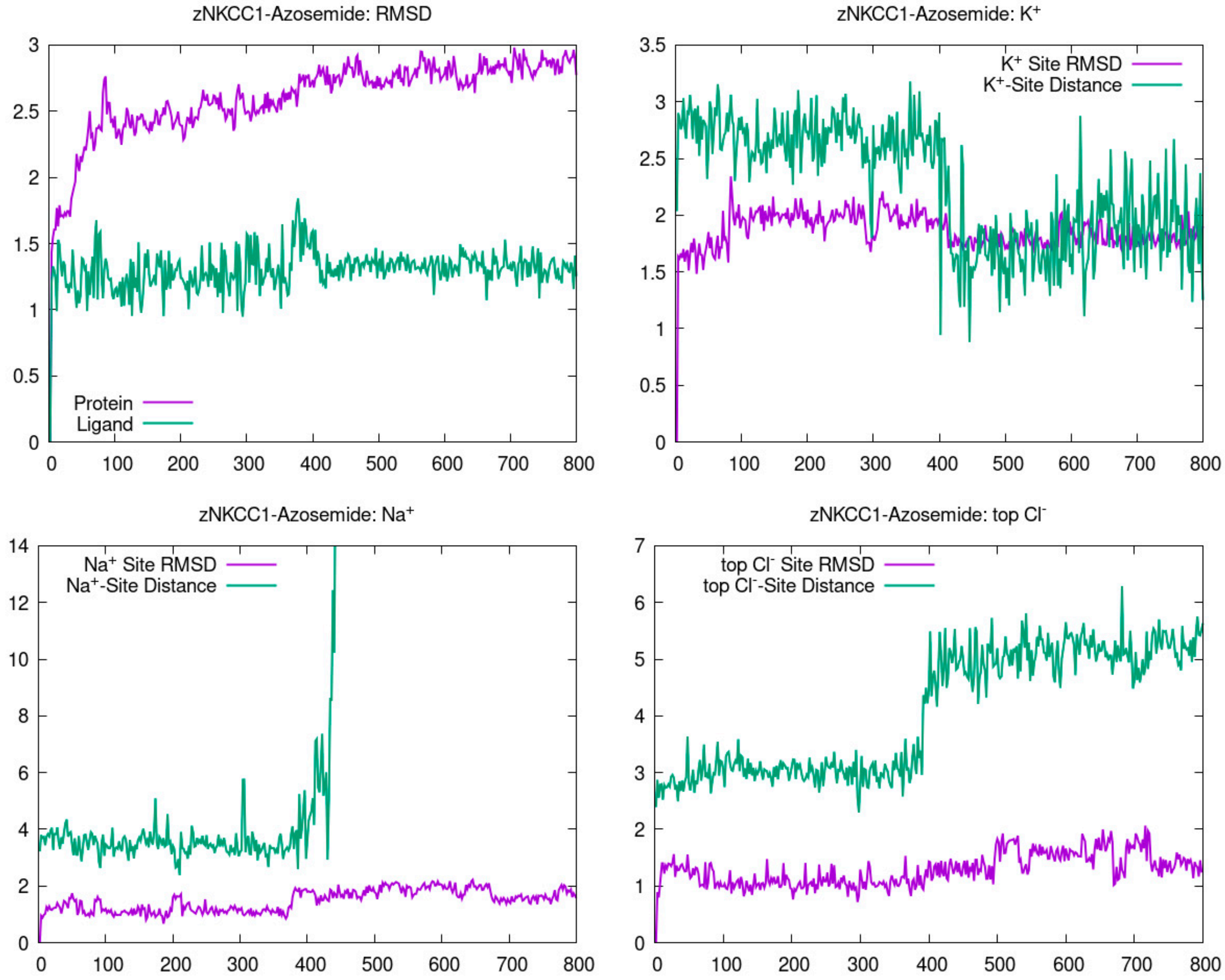

Figure S9: Root mean square deviation (RMSD, Å) of the azosemide/zebrafish NKCC1 complex for the protein (violet line, top left) and drug (green line, top left). RMSD of the ion binding sites with respect to the first frame of the simulation (violet lines) and distance of the ion with respect to the center of its ion binding site (green lines) vs simulation time (ns) for K^+ (top right), Na^+ (bottom left), the top Cl^- (bottom right).

Table S5. Molecular Mechanics Poisson Boltzmann Surface Area (MM/PBSA) binding free energies (ΔG_b, kcal/mol) per residue as obtained by the alanine scanning of the zebrafish NKCC1/azosemide complex. The relative binding free energies ($\Delta\Delta G_b$) are calculated as ΔG_b of mutant - ΔG_b of wild type. The most important residues (with $\Delta\Delta G_b > 0.6$ kcal/mol) are highlighted in bold. Residues identified by mutagenesis studies as important for the bumetanide inhibition are marked with *. Values from the MM/PBSA calculation in the absence of one of the ions are also provided to evaluate the importance of the ions for the azosemide binding.

	$\Delta G_{binding}$ (kcal/mol)	Std. Dev.	Std. Err. of Mean	$\Delta\Delta G_{binding}$ (kcal/mol)
WT	-27.59	3.32	0.55	
**N220A**	**-25.90**	3.26	0.54	**1.69**
V224A	-27.25	3.30	0.55	0.34
M225A	-27.45	3.33	0.55	0.14
I293A*	-27.59	3.32	0.55	0.00
**F294A***	**-24.80**	3.13	0.52	**2.79**
**N298A**	**-24.91**	3.58	0.60	**2.68**
V300A*	-27.57	3.31	0.55	0.03
V302A	-27.57	3.31	0.55	0.02
**M304A***	**-25.62**	3.22	0.54	**1.97**
**Y305A**	**-23.90**	3.08	0.51	**3.69**
**T420A**	**-24.96**	3.33	0.55	**2.63**
L423A	-27.18	3.34	0.56	0.41
N427A	-27.30	3.26	0.54	0.29
S539A	-27.49	3.31	0.55	0.10
S543A	-27.06	3.34	0.56	0.53
S546A	-27.23	3.35	0.56	0.36
V550A	-27.34	3.32	0.55	0.25
L596A	-27.60	3.32	0.55	0.00
**I603A**	**-25.39**	3.35	0.56	**2.20**
F607A	-27.18	3.37	0.56	0.41
S610A	-27.54	3.31	0.55	0.05
ΔK^+	-24.53	3.21	0.54	3.06
ΔCl^-	-28.26	3.19	0.53	-0.67

Furosemide binding to zebrafish NKCC1

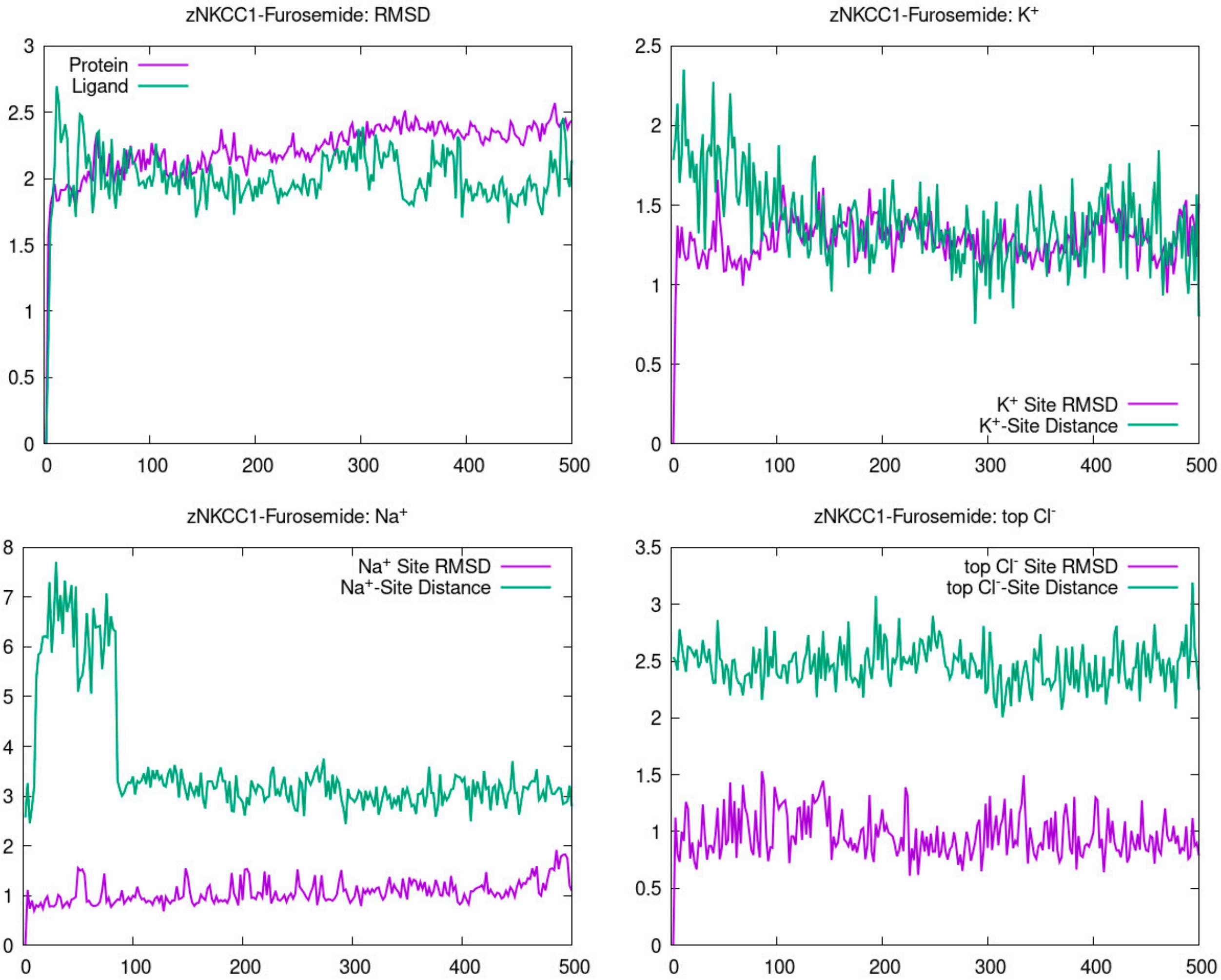

Figure S10. Root mean square deviation (RMSD, Å) of the furosemide/zebrafish NKCC1 complex for the protein (violet line, top left) and drug (green line, top left). RMSD of the ion binding sites with respect to the first frame of the simulation (violet lines) and distance of the ion with respect to the center of its ion binding site (green lines) vs simulation time (ns) for K^+ (top right), Na^+ (bottom left), the top Cl^- (bottom right).

Table S6. Molecular Mechanics Poisson Boltzmann Surface Area (MM/PBSA) binding free energies (ΔG_b, kcal/mol per residue as obtained by the alanine scanning of the zebrafish NKCC1/furosemide complex. The relative binding free energies ($\Delta\Delta G_b$) are calculated as ΔG_b of mutant - ΔG_b of wild type. The most important residues (with $\Delta\Delta G_b$ > 0.6 kcal/mol) are highlighted in bold. Residues identified by mutagenesis studes as important for the bumetanide inhibition are marked with *. Values from the MM/PBSA calculation in the absence of one of the ions are also provided to evaluate the importance of the ions for the furosemide binding.

	$\Delta G_{binding}$ (kcal/mol)	Std. Dev.	Std. Err. of Mean	$\Delta\Delta G_{binding}$ (kcal/mol)
WT	-24.80	4.34	0.87	
**N220A**	**-24.10**	4.36	0.87	**0.70**
I221A	-24.75	4.34	0.87	0.04
V224A	-24.52	4.40	0.88	0.27
M225A	-24.76	4.34	0.87	0.04
I293A*	-24.79	4.34	0.87	0.00
**F294A***	**-23.92**	4.37	0.87	**0.88**
**N298A**	**-24.11**	4.42	0.88	**0.69**
V300A*	-24.78	4.34	0.87	0.01
**M304A***	**-24.11**	4.29	0.86	**0.69**
**Y305A**	**-22.81**	4.33	0.87	**1.99**
**T420A**	**-23.60**	4.44	0.89	**1.20**
I422A	-24.58	4.32	0.86	0.21
**L423A**	**-23.82**	4.25	0.85	**0.97**
N427A	-24.45	4.26	0.85	0.35
S546A	-24.66	4.35	0.87	0.14
L596A	-24.79	4.34	0.87	0.00
**I603A**	**-23.92**	4.26	0.85	**0.87**
**F607A**	**-21.83**	4.43	0.89	**2.96**
**Y611A**	**-23.62**	4.35	0.87	**1.17**
Na^+	-22.57	4.25	0.85	2.23
K^+	-20.93	4.08	0.82	3.86
Cl^-	-25.68	4.39	0.88	-0.89

Docked poses of azosemide, furosemide and ethacrynic acid to NKCC1

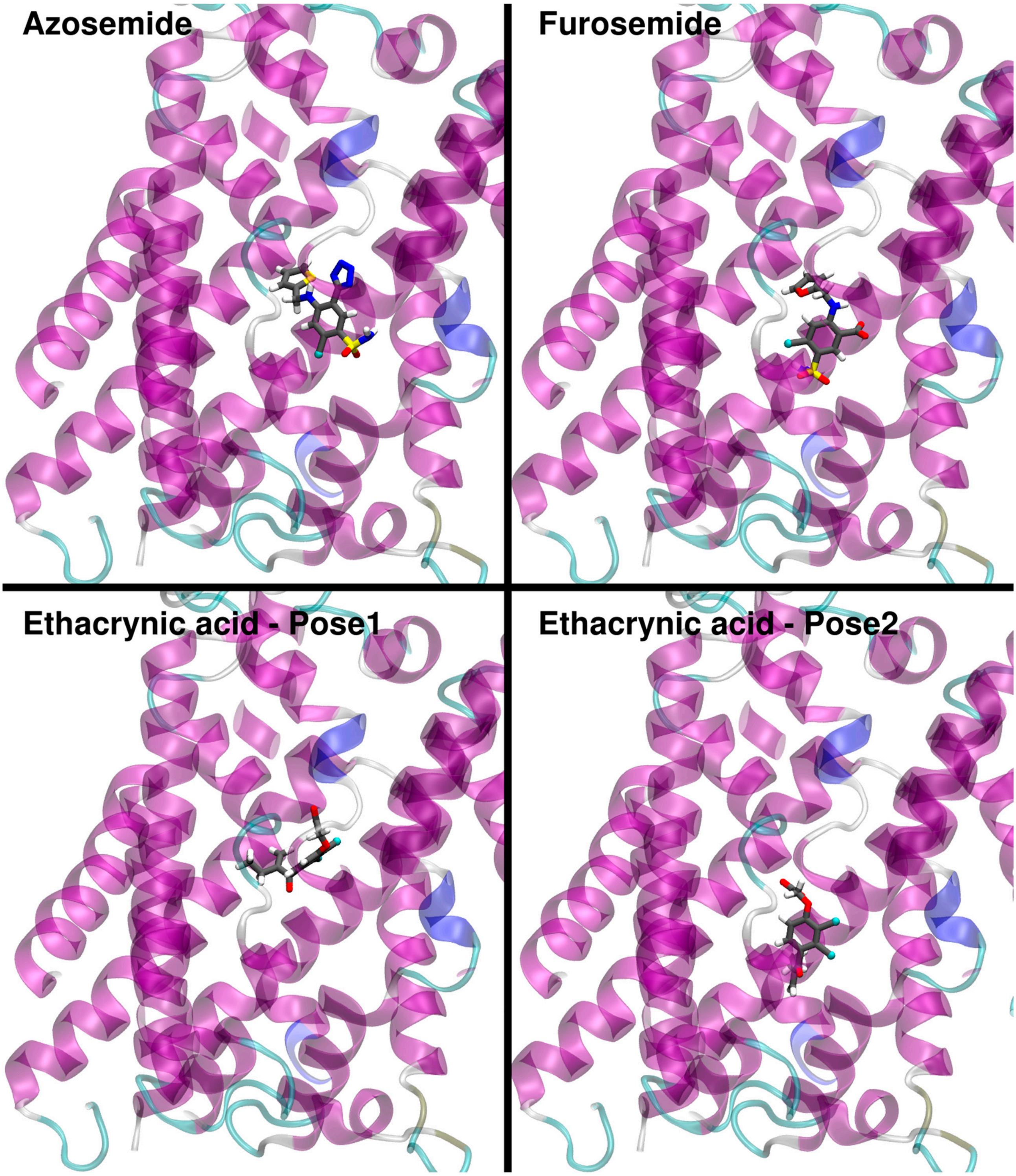

Figure S11. The initial binding poses of azosemide, furosemide and ethacrynic acid to the inward facing conformation of the zebrafish NKCC1 model. NKCC1 is depicted as magenta new cartoons with loops depicted in cyan. The drugs are shown in licorice and colored by atom name. The binding enthalpies (calculated with MM/PBSA) of the two ethacrynic acid poses were -22.3 ± 3.7 kcal/mol and -18.2 ± 3.9 kcal/mol for Pose1 and Pose2, respectively.

Ethacrynic acid binding to zebrafish NKCC1

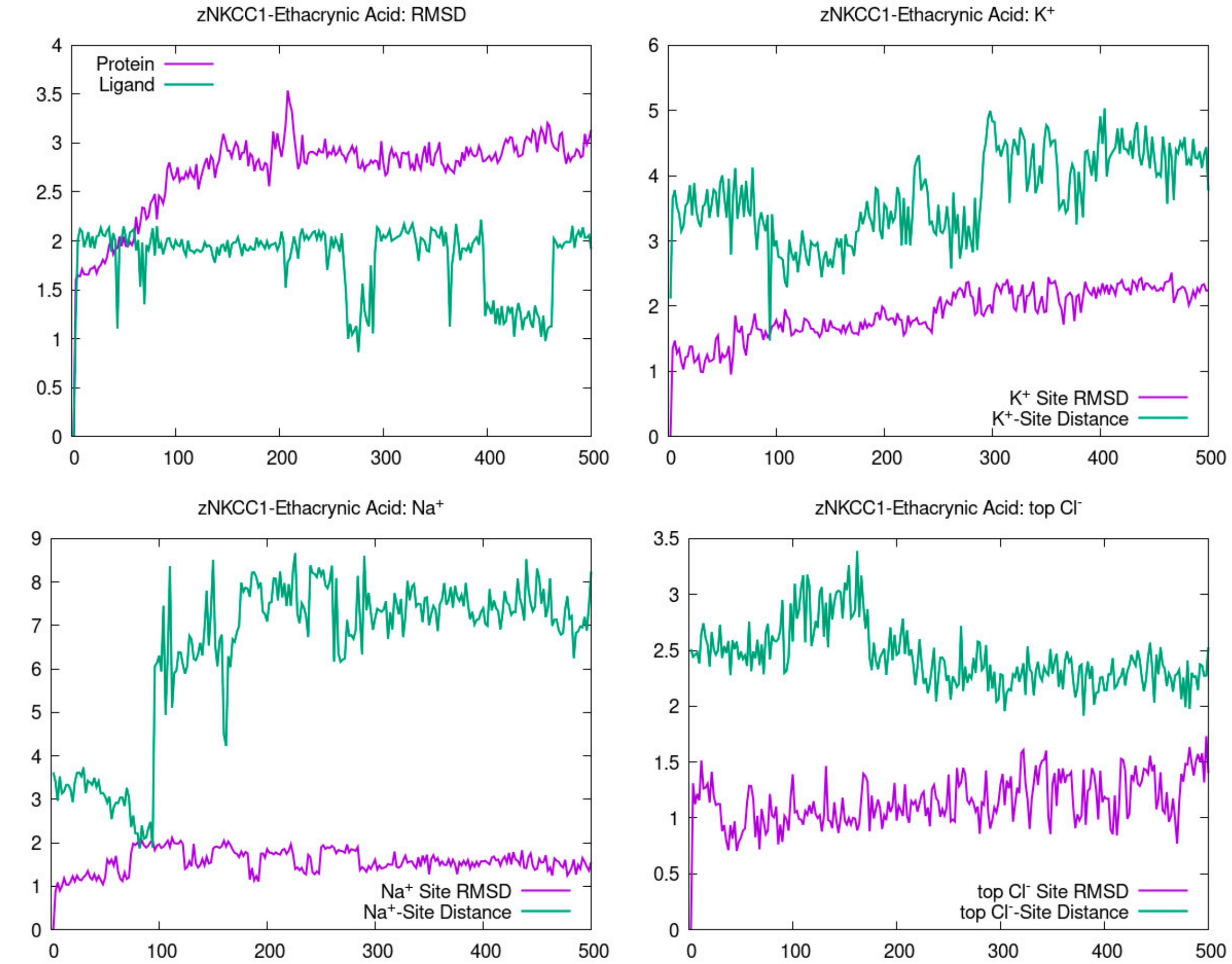

Figure S12. Root mean square deviation (RMSD, Å) of the ethacrynic acid/zebrafish NKCC1 complex for the protein (violet line, top left) and drug (green line, top left). RMSD of the ion binding sites with respect to the first frame of the simulation (violet lines) and distance of the ion with respect to the center of its ion binding site (green lines) vs simulation time (ns) for K^+ (top right), Na^+ (bottom left), the top Cl^- (bottom right).

Table S7. Molecular Mechanics Poisson Boltzmann Surface Area (MM/PBSA) binding free energies (ΔG_b, kcal/mol) per residue as obtained by the alanine scanning of the zebrafish NKCC1/ethacrynic acid complex. The relative binding free energies ($\Delta\Delta G_b$) are calculated as ΔG_b of mutant - ΔG_b of wild type. The most important residues (with $\Delta\Delta G_b > 0.6$ kcal/mol) are highlighted in bold. Residues found by mutagenesis studies as important for the bumetanide inhibition are marked with *. Values from the MM/PBSA calculation in the absence of one of the ions are also provided to evaluate the importance of the ions for the ethacrynic acid binding.

	$\Delta G_{binding}$ (kcal/mol)	Std. Dev.	Std. Err. of Mean	$\Delta\Delta G_{binding}$ (kcal/mol)
WT	-22.11	3.64	0.73	
W222A	-22.09	3.64	0.73	0.01
**V224A**	**-20.62**	3.48	0.70	**1.49**
M225A	-21.93	3.62	0.72	0.18
L226A	-22.12	3.64	0.73	-0.01
I228A	-21.97	3.66	0.73	0.14
I293A*	-22.10	3.64	0.73	0.00
F294A*	-22.10	3.64	0.73	0.01
V300A*	-22.06	3.67	0.73	0.04
V302A	-22.10	3.64	0.73	0.00
**M304A***	**-20.83**	3.80	0.76	**1.28**
**Y305A**	**-20.19**	3.55	0.71	**1.91**
S413A	-22.12	3.64	0.73	-0.01
I414A	-22.09	3.62	0.72	0.01
**F416A**	**-21.30**	3.65	0.73	**0.80**
**T420A**	**-21.44**	3.65	0.73	**0.67**
S536A	-21.91	3.59	0.72	0.20
**L596A**	**-20.36**	3.76	0.75	**1.74**
I599A	-21.68	3.61	0.72	0.42
**I603A**	**-21.32**	3.68	0.74	**0.79**
S604A	-21.84	3.65	0.73	0.27
**F607A**	**-21.32**	3.62	0.72	**0.78**
Na^+	-20.26	3.57	0.71	1.85
K^+	-19.57	3.36	0.67	2.53
Cl^-	-24.04	3.73	0.75	-1.94

Bumetanide binding to the outward-facing conformations of hNKCC1

Table S8. Binding enthalpy (ΔH_b) of BUM binding to the outward-facing conformation of hNKCC1 (hNKCC1$_{outward}$) extracted from MM/PBSA calculations. Structure of BUM bound to the outward-facing conformation of hNKCC1 was taken from PDB ID: 7S1X [42]. The mutations introduced to capture the conformation were reversed to wild type. The system was built using the same force field setup as described in main text. The system was not placed into a membrane or solvated. Instead, eight water molecules were placed in vicinity of BUM and/or K^+ ion (to be consistent with our MM/PBSA protocol) and the system minimized in 1000000 steps of steepest-decent optimization in implicit solvent with restraints on the protein backbone. The single optimized frame was then used to calculate 'single-point' MM/PBSA ΔH_b following the same 'membrane-corrected' protocol as described in main text in order to obtain approximate/qualitative estimation of BUM binding to hNKCC1$_{outward}$. For comparison purposes, same 'single-point' MM/PBSA calculation was done also for the representative structure of the BUM binding to the inward-facing conformation of hNKCC1 (hNKCC1$_{inward}$) that we obtained in our study. Full trajectory ΔH_b of hNKCC1$_{inward}$-BUM is provided as well. Entropic contribution to binding was not considered in this analysis.

	ΔH_b – 'single-point' [kcal/mol]	ΔH_b – 'full trajectory' [kcal/mol]
hNKCC1$_{inward}$ – BUM	-30.3	-
hNKCC1$_{outward}$ – BUM	-33.3	-32.6 ± 3.4

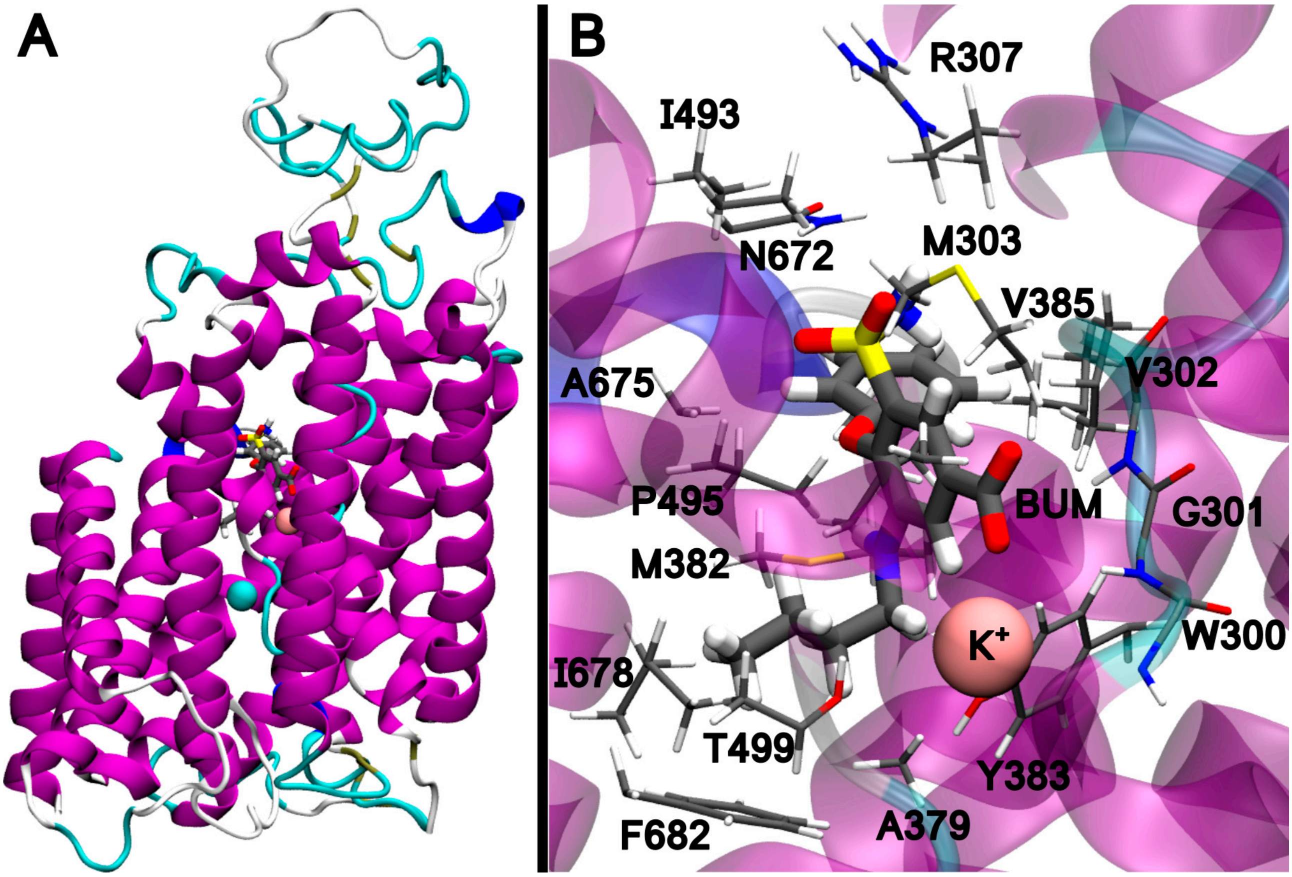

Figure S13. Binding mode of bumetanide (BUM) to the outward-facing conformation of hNKCC1 (PDB ID: 7S1X). Overall (A) and close up (B) view of the BUM binding mode to the outward facing conformation of hNKCC1. NKCC1 is depicted as magenta, yellow, blue, cyan and white new cartoons for α-helix, β-sheet, 3_{10}-helix, turn and coil elements, respectively. The Cl^- and K^+ ions are shown as cyan and orange van der Waals spheres, respectively. The BUM and important NKCC1 residues are shown in thick and thin licorice, respectively, and colored by atom name.

Inner cavity definition

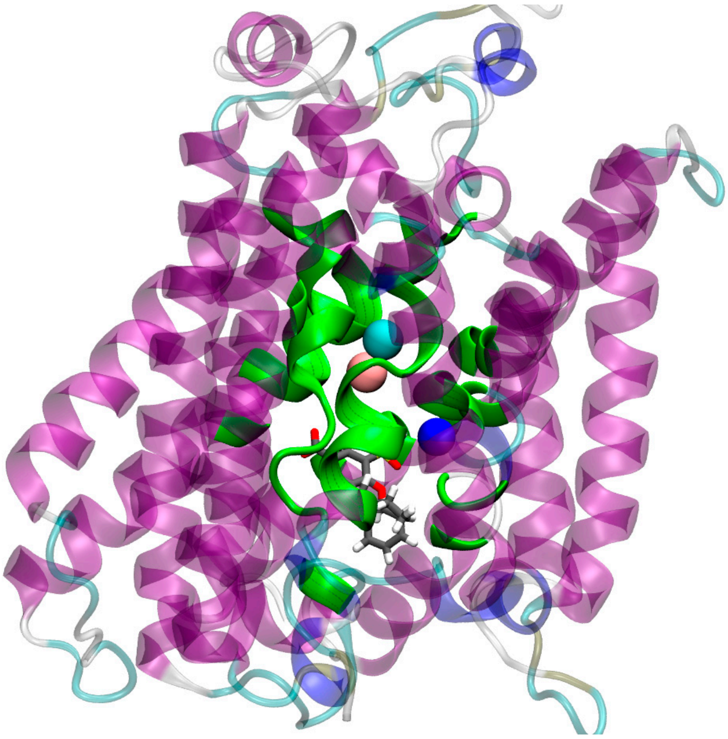

Figure S14. NKCC1 inner cavity definition used to calculated the number of contacts between bumetadine (BUM) and zNKCC1 for the purpose of reweighting the metadynamics free energy surface. NKCC1 is shown as magenta new cartoons with loops depiced in cyan, BUM as licorice and the Cl^{-}, Na^{+}, and K^{+} ions as cyan, blue, and orange van der Waals spheres. The inner cavity is highlighted in green and is composed of following residues: 216-225, 297, 298, 300-305, 364, 413, 414, 416-424, 427, 454, 532, 535, 536, 542, 543, 546, 596, 600, 602-608, 611.
